# Leucine-enriched amino acid supplementation and exercise to prevent sarcopenia in patients on hemodialysis: a single-arm pilot study

**DOI:** 10.3389/fnut.2023.1069651

**Published:** 2023-04-28

**Authors:** Sang-Hyeon Ju, Eu Jin Lee, Byeong Chang Sim, Ha Thi Nga, Ho Yeop Lee, Jingwen Tian, Kyu Jeong Cho, Hyoungsu Park, Dae Eun Choi, Young Rok Ham, Hyon-Seung Yi

**Affiliations:** ^1^Department of Internal Medicine, Chungnam National University School of Medicine, Daejeon, Republic of Korea; ^2^Laboratory of Endocrinology and Immune System, Chungnam National University School of Medicine, Daejeon, Republic of Korea; ^3^Department of Medical Science, Chungnam National University School of Medicine, Daejeon, Republic of Korea; ^4^R&D Group, Maeil Health Nutrition Co., Ltd., Pyeongtaek, Republic of Korea

**Keywords:** sarcopenia, hemodialysis, leucine, protein supplementation, exercise

## Abstract

**Background:**

Sarcopenia, which is strongly associated with mortality and quality of life, occurs in up to 40% of hemodialysis patients. Here, we investigated the preventive effects of leucine-enriched amino acid supplementation and resistance exercise in non-sarcopenic hemodialysis patients, and characterized the biochemical and immunophenotypic profiles of those who benefited from the intervention.

**Methods:**

Twenty-two patients on maintenance hemodialysis at our hospital were enrolled in this single center, prospective, single-arm pilot trial. For the first 12 weeks, the subjects were administered a total of 6 g of leucine per day. Three grams were supplied via capsules, and the remaining three grams were provided via beverages containing macro- and micro- nutrients, such as 10 μg of vitamin D and 290 mg of calcium. The supplements were not provided for the next 12 weeks. Muscle mass, grip strength, and physical performance were measured using the bioimpedance analyzer (BIA), handgrip strength (HGS), and short physical performance battery (SPPB) protocols, respectively, at baseline, 12 weeks, and 24 weeks. In addition, serum biochemistry, immunophenotype of peripheral blood mononuclear cells, and nutritional status was assessed at the three time points. Those who showed 5% or more improvement in parameters were defined as responders, otherwise, as non-responders (ClinicalTrials.gov identification number: NCT04927208).

**Results:**

Twenty-one out of twenty-two patients (95.4%) showed improvement in at least one or more parameters among muscle mass, grip strength, and physical performance. After 12 weeks of intervention, skeletal muscle index was increased in 14 patients (63.6%), and grip strength was improved in 7 patients (31.8%). Baseline grip strength lower than 35.0 kg was the strongest predictor of improvement in grip strength (AUC 0.933 from ROC curve). Grip strength showed a significant increase in females than males (7.6 ± 8.2 vs. −1.6 ± 7.2%, *p* = 0.03), in age over 60 than under 60 (5.3 ± 6.2 vs. −1.4 ± 9.1%, *p* = 0.04), and in higher (≥95%) than lower (<95%) exercise compliance (6.8 ± 7.7 vs. −3.2 ± 6.4%, *p* = 0.004). In SPPB study, gait speed and sit-to-stand time was improved in 13 patients (59.1%) and 14 patients (63.6%), respectively. Baseline hemoglobin lower than 10.5 g/dl and hematocrit lower than 30.8% were predictor of improvement in the sit-to-stand time (AUC 0.862 and 0.848, respectively). Serum biochemistry results showed that, compared to non-responders, responders in muscle mass had lower baseline monocyte fraction (8.4 ± 1.9 vs. 6.9 ± 1.1%, *p* = 0.03), and responders in grip strength had lower baseline total protein (6.7 ± 0.4 vs. 6.4 ± 0.3 g/dL, p = 0.04). Immunophenotypic analysis found that the intervention tended to increase the naïve/memory CD8+ T cell ratio (from 1.2 ± 0.8 to 1.4 ± 1.1, p = 0.07).

**Conclusion:**

Leucine-enriched amino acid supplementation and resistance exercise induced significant improvement in muscle mass, strength, and physical function in subpopulation of the non-sarcopenic hemodialysis patients. Those who benefited from the intervention were old-age females with lower baseline grip strength or lower hemoglobin or hematocrit, and who have good exercise compliance. Therefore, we propose that the intervention will help to prevent sarcopenia in selected patients on maintenance hemodialysis.

## Introduction

Hemodialysis (HD) patients are less active, due primarily to reduced exercise capacity and poor physical performance resulting from muscle abnormalities (1, 2). The clinical outcome of muscle abnormalities is sarcopenia, which is defined as age-related loss of muscle mass plus low muscle strength and/or low physical performance (3). In the 2019 consensus update, the Asian Working Group for Sarcopenia proposed diagnostic criteria for sarcopenia as having low appendicular skeletal muscle mass (ASM) plus low handgrip strength (HGS) or low physical performance (3). A systematic review reported a sarcopenia prevalence of 28.5% in dialysis patients (4). Focusing on HD, the prevalence of sarcopenia has been reported as 13.7–42.4% in articles using European Working Group on Sarcopenia in Older People (EWGSOP) criteria and 36–40% in articles using Asian Working Group for Sarcopenia (AWGS) criteria (5). The high prevalence of sarcopenia in HD patients compared to the general population, where the prevalence of sarcopenia is 10%, can be attributed to several reasons (6). Abnormal muscle architecture and function are more prominent in HD patients than in patients with chronic kidney disease (CKD) not yet on HD (7, 8). Moreover, factors related to CKD or HD, such as uremic neuropathy, abnormal vitamin D metabolism, acidosis, hyperparathyroidism, and malnutrition, may contribute to muscle loss and weakness (9). CKD is also associated with chronic inflammation, leading to catabolic destruction of structural and functional proteins, muscle proteolysis, and loss of the ability to exercise (9). Therefore, evaluation of muscle function in patients receiving HD is a critical component of clinical performance measurement (10).

Malnutrition caused by a deficit in protein and calories is common in HD patients, resulting in poor nutritional status and increased morbidity and mortality (11–13). Patients receiving HD exhibit a negative protein and calorie balance due to inevitable loss of amino acids into the hemodialysate when coupled to conventional dialyzers or high-flux dialyzers (approximately 1–2 g or 6–12 g is lost, respectively) (14, 15). In addition, caspase-3-mediated apoptosis and activation of the ubiquitin-proteasome system accelerate CKD-induced muscle proteolysis and wasting (16, 17). Furthermore, HD patients display “anabolic resistance” to physical exercise and dietary protein/amino acids (18–20). Although the combination of physical exercise and nutritional supplementation may provide an anabolic stimulus in HD patients, it is not clear if and to what extent amino acids or physical exercise trigger anabolic effects in HD patients.

Systemic inflammation is closely associated with atherosclerosis, coronary artery disease, and sarcopenia, which are the major causes of morbidity and mortality in HD patients. In fact, chronic inflammatory responses, as evaluated by acute phase C-reactive protein, cytokine, and chemokine levels in the peripheral blood of CKD patients, increase with deteriorating renal function (21). Moreover, HD patients have lower CD4+ and CD8+ T cell counts in peripheral blood than CKD patients not on HD (22). In addition, peripheral blood mononuclear cells (PBMCs) of HD patients show higher expression of Fas and FasL, which may initiate apoptosis by activating the caspase cascade (23). However, the relationship between the type of systemic immune cell and muscle mass and strength, or the ability of nutritional and exercise interventions to improve muscle dysfunction, in HD patients remains unclear.

In light of these unanswered questions, our study has three aims: (1) to evaluate the effect of leucine-enriched amino acid supplementation and resistance exercise on muscle mass and function in HD patients, (2) to investigate the baseline characteristics of patients who benefit from the intervention, and (3) to examine changes in serum biochemistry and immune cell proportion due to the intervention. Here, we recruited 22 HD patients to a single center, prospective, single-arm interventional pilot trial of leucine-enriched amino acid supplementation and resistance exercise. Our results provide insight into the potential contributions of leucine supplementation and exercise on prevention/reversal of muscle weakness in HD patients.

## Materials and methods

### Subjects and eligibility criteria

This is a single center, prospective, single arm pilot study performed at the Chungnam National University Hospital, Daejeon, Republic of Korea between January 2021 and September 2021. The inclusion criteria were as follows: maintenance hemodialysis patients (MHD; patients had HD three sessions per week and each session lasted 4 h which was prescribed by the nephrologist in the division of nephrology) in our hospital; MHD duration more than 6 months; completely independent in Barthel index for activities of daily living; 20–80 years of age. The exclusion criteria were as follows: a history of ongoing cancer or a cancer-free period <5 years; psychiatric disease; malabsorption disorder (including diabetic gastropathy or a history of bowel resection); pregnancy; acute bacterial or viral infection; or requirement of a wheelchair for mobility.

### Ethical considerations

All participants received information about the goals and procedures of the study, and all agreed to participate by signing a consent form. The protocol was reviewed and approved by the Institutional Review Boards of Chungnam National University Hospital (CNUH-2020-10-019-005). All ongoing and related trials for this intervention are registered at the Clinicaltrials.gov website (NCT04927208).

### Outcome measurement

Primary endpoint:

1. Improvement of muscle mass, strength, and physical performance after 12 weeks of leucine-enriched amino acid supplementation and resistance exercise.

Secondary endpoint:

1. Effect of leucine-enriched amino acid supplementation and resistance exercise on serum biochemistry.
2. Effect of leucine-enriched amino acid supplementation and resistance exercise on immunophenotype of peripheral blood mononuclear cells.
3. Nutritional status improvement after 12 weeks of leucine-enriched amino acid supplementation and resistance exercise.

### Study protocol

**FIGURE 1:**
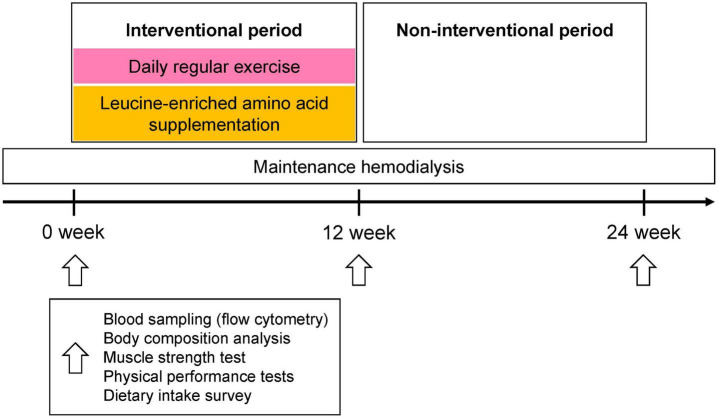
Study protocol. For the first 12 weeks of the intervention period, all participants (N = 22) were given 6 g of leucine containing supplements (125 ml thrice daily plus three 333 mg capsules thrice daily) and performed daily exercise (exercise compliance, mean ± SD, 87.1 ± 15.3%). During the next 12 weeks, participants neither drank the beverage nor exercised regularly. Blood sampling, bioimpedance analysis, muscle strength, and physical performance tests, as well as monitoring of dietary intake, were performed at baseline and at 12 and 24 weeks. Blood sampling, muscle strength test, and physical performance tests were performed immediately before HD session. Body composition test was performed pre- and post- HD, and post-HD body composition was used for analysis.

Patients received a leucine-enriched amino acid beverage plus leucine capsules in advance of the study so that supplementation had begun by the first day of intervention. Baseline blood sampling, body composition analysis, muscle strength test, and physical performance tests were performed at the last visit for HD, prior to the supplementation and exercise intervention. During the first 12 weeks of intervention, exercise compliance, supplementation-related gastrointestinal symptoms (dyspepsia, abdominal bloating, and diarrhea), and exercise related adverse events were monitored weekly. At the end of the 12 weeks of intervention period, and after another 12 weeks of non-intervention period, blood sampling, body composition analysis, muscle strength test, and physical performance tests were repeated using the same methods used for the baseline tests. The muscle strength and physical function tests were performed 1–2 h before HD. In addition, a blood sample was drawn immediately after HD. Body composition was measured after HD. To assess nutritional status and dietary habits, we used 24-h recall methods at baseline, 12 weeks, and 24 weeks (Figure 1). In evaluating the efficacy of intervention, responders were defined as individuals who showed a 5% improvement either in muscle strength or each physical function test, considering test and retest reliability (24, 25). For muscle mass, any increase was considered sufficient to meet the definition of a responder.

### Study parameters

We analyzed parameters to assess components of the sarcopenia including appendicular skeletal muscle mass (ASM), HGS, and short physical performance battery (SPPB). To evaluate body composition before and after the intervention, patients underwent bioelectrical impedance analysis (BIA), and the data included not only ASM but also fat mass. In addition, serum biochemistry parameters were analyzed; liver function (AST, ALT, ALP, total bilirubin, total protein, and albumin, and glucose), renal function (BUN, Cr, eGFR, and uric acid), electrolytes (Ca, P, Na, K, Cl), lipid profile (total cholesterol, triglyceride, HDL, LDL), blood cell count (WBC, RBC, platelet, neutrophil, lymphocyte, monocyte, eosinophil, and basophil), detailed blood cell parameters (hemoglobin, hematocrit, mean corpuscular volume, mean corpuscular hemoglobin, mean corpuscular hemoglobin concentration, and mean platelet volume), and CRP. Immunophenotypic analysis of peripheral blood mononuclear cells (PBMC) evaluated percentage of immune cell population by expression of surface markers; CD4, CD8, CD57, CD14, CD16, CD3, CD56, CD45RA, CD45RO, CD197, CD279, FOXP3, TNFα, IL17A, and γδTCR. We also used clinical parameters of compliances for exercise or leucine supplement and dialysis adequacy using Kt/V.

### Exercise intervention

We provided an exercise protocol to our patients and instructed them to exercise daily. The exercise intervention includes stretching and full body exercises and it takes about 1 hour. The full body exercise consist of arm exercises using dumbbells or a water bottle, leg exercises using a chair, and daily walking. Exercise compliance was assessed weekly when the participants visited the hospital for HD. More specific information about the exercise protocol is provided in the Supplementary material (Supplementary PDF).

### Leucine-enriched amino acid supplementation

The subjects received a daily total supplementation of 6 g of leucine, with half provided in the form of a beverage and the other half in capsules. The subjects received a 125 ml protein-supplemented beverage (Maeil Health Nutrition Co., Ltd., Gyeonggi-do, Korea) containing 8 g of protein, 1 g of total leucine, 10 μg (400 IU) of vitamin D, and 290 mg of calcium three times a day. Detailed information about the beverage can be found in Supplementary Table 1. In addition, participants were also provided with leucine-enriched capsules (333 mg × 3 capsules, three times a day), which supplied half of the daily total supplementation of 6 g of leucine. All beverages were grain-flavored to mask the contents and were stored at room temperature. The subjects consumed one beverage and three capsules immediately after each meal.

### Nutritional status assessment using 24 h dietary recall method

The 24-h dietary recall method was used to monitor dietary intake. At baseline, 12 weeks, and 24 weeks, patients were asked to recall the type and amount of all food and beverages consumed in the last 24 hours. This data was uploaded to CAN-Pro software (ver. 5.0, 2015; The Korean Nutrition Society, Seoul, Republic of Korea) to analyze the average amount of nutrients consumed. Using the software, we were able to quantify both macro- and micro- nutrients including vitamins and minerals.

### Assessment of sarcopenia

Body composition analysis was performed using a bioimpedance analyzer (InBody 270 Body Composition Analyzer; InBody USA, Cerritos, CA, USA). Appendicular skeletal muscle mass (ASM) was defined as the sum of the muscle mass of all four limbs. The skeletal muscle index (SMI) was defined as appendicular skeletal muscle mass divided by height squared (ASM/m^2^).

Muscle strength was examined by measuring HGS. HGS of the non-HD access (arteriovenous fistula or graft) side was measured using a Smedley type dynamometer (Takei T.K.K.5401 GRIP-D handgrip dynamometer; Takei Scientific Instruments Co., Ltd, Tokyo, Japan). Participants were instructed to adopt a comfortable sitting position, bend their elbows to 90^°^ (90^°^ flexion), and squeeze the dynamometer as hard as possible. The test was duplicated three times at 1 min intervals, and the maximum value was recorded.

Physical performance was evaluated using the SPPB, which includes standardized performance tests: gait speed in a 4-m walk test; a five times sit-to stand test (5TSTS) of coordination and strength; and a tandem test for static balance. The tandem test was performed in three different positions: a side-by-side position, a semi-tandem position, and a full tandem position. The patients were asked to maintain each position for >10 s, and the amount of time (s) that they successfully remained in the given position was recorded.

A diagnosis of sarcopenia was based on the 2019 Consensus Guidelines from the Asian Working Group for Sarcopenia (3). Patients with low appendicular muscle mass (ASM measured by BIA: <7.0 kg/m^2^ for males and <5.7 kg/m^2^ for females) and low muscle strength (HGS <28 kg for males and <18 kg for females) with or without low physical performance (gait speed <1.0 m/s; 5TSTS ≥12 s; or SPPB score ≤9 points) were classified as sarcopenic.

### Isolation of PBMCs

Peripheral blood samples were obtained from all study participants, transferred aseptically into 50 ml polystyrene centrifuge tubes containing ethylenediaminetetraacetic acid (Sigma-Aldrich, Dorset, UK) as an anticoagulant, and gently mixed. Serum samples were prepared by centrifugation at 2,000 × *g* for 10 min at 4°C; PBMCs were isolated by centrifugation on a Ficoll-Paque density gradient (GE Healthcare Life Science, Buckinghamshire, UK) at room temperature. After centrifugation, the PBMC layer was collected and washed in Dulbecco’s phosphate-buffered saline. The isolated and washed PBMCs were resuspended in 2 ml of Roswell Park Memorial Institute 1640 medium (Welgene, Daegu, South Korea), and tested by trypan blue dye exclusion to determine the number of viable cells. Samples were stained for flow cytometry analyses using fluorescence-conjugated monoclonal antibodies.

### Flow cytometry analysis

PBMCs were pre-incubated with an anti-mouse CD16/32 Fc blocker (BD Pharmingen, USA), followed by anti-FVD-APC-Cy7 (all supplied by eBioscience, San Diego, CA, USA) to exclude dead cells. After washing with FACS staining buffer, cells were treated with fluorochrome-conjugated monoclonal antibodies for 40 min at 4°C. The monoclonal antibodies used in this study were as follows: anti-CD3-PerCP-Cy5.5, anti-CD3-PE-Cy7, anti-CD4-AF700, anti-CD8-PE, anti-CD8-APC, anti-CD28-APC, anti-CD45RA-FITC, anti-CD45RO-PE-Cy7, anti-CD57-FITC, anti-TCR gamma/delta-FITC, fixable viability dye-APC-Cy7, anti-interferon (IFN)-γ-PE-Cy7, and anti-tumor necrosis factor (TNF)-α-APC (all supplied by eBioscience, San Diego, CA, USA). For intracellular staining, surface-stained cells were stimulated with phorbol-myristate acetate/ionomycin/brefeldin A/monensin for 5 h, and then fixed and permeabilized using a Fixation/Permeabilization Buffer kit (eBioscience, San Diego, CA, USA). The permeabilized cells were washed and resuspended in 1% formaldehyde and stained with anti-IFN-γ-PE-Cy7 and anti-TNF-α-APC. Multicolor flow cytometry was performed using a BD LSRFortessa flow cytometer (BD Biosciences, San Jose, CA, USA), and the data were analyzed by FlowJo V10 software (FlowJo, LLC, Ashland, OR, USA).

### Serum chemistry

Peripheral blood was collected into heparin-coated tubes. Plasma levels of TSH, T3, free T4, and TBII were measured by an automated analyzer (Cobas 6000; Roche Diagnostics GmbH, Mannheim, Germany). Plasma lipid profiles, including low-density lipoprotein cholesterol, high-density lipoprotein cholesterol, total cholesterol, triglycerides, and creatine kinase, were evaluated using a blood chemistry analyzer (Hitachi 47; Hitachi, Tokyo, Japan). Aspartate transaminase and alanine transaminase activities were measured using the International Federation of Clinical Chemistry Ultra Violet method, without pyridoxal phosphate (TBA-2000FR; Toshiba, Tokyo, Japan).

### Sample size and statistical analysis

**FIGURE 2:**
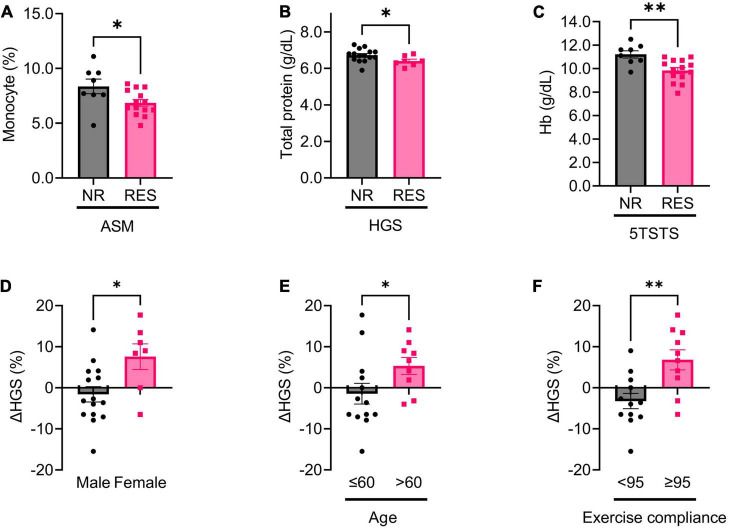
Baseline, and changes in, biochemical parameters in non-responders (NR) and responders (RES) during the first 12 weeks of the intervention period. **(A)** Baseline monocyte fraction in appendicular skeletal muscle mass (ASM) NR and RES group. **(B)** Baseline total protein in handgrip strength (HGS) NR and RES group. **(C)** Baseline hemoglobin in 5 times sit-to stand test (5TSTS) NR and RES group. **(D–F)** Changes in handgrip strength during the intervention period according to sex **(D)**, age **(E)**, and exercise compliance **(F)**. Unpaired *t*-test for panels **(A–F)**. NR, non-responder; RES, responder; ASM, appendicular skeletal muscle mass; HGS, handgrip strength; Hb, hemoglobin. *P < 0.05, **P < 0.01. Data are presented as the mean ± SEM.

**FIGURE 3:**
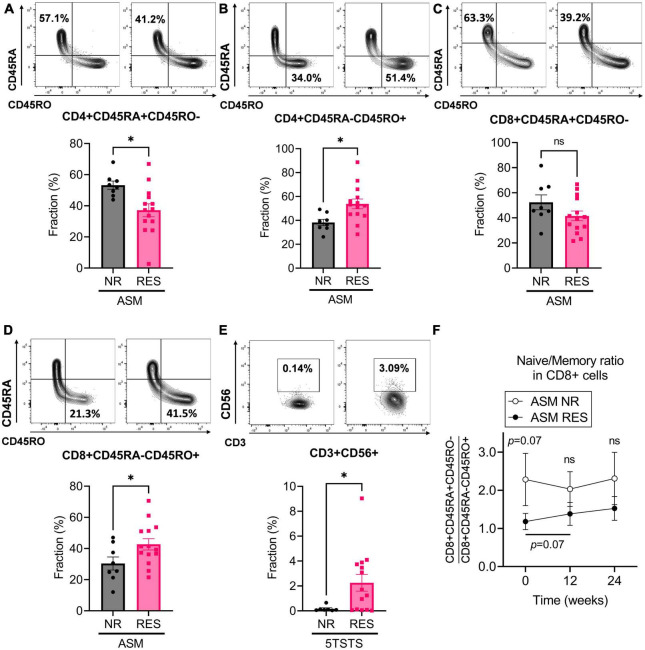
Baseline immunophenotypic characteristics of patients by ASM and 5TSTS results. **(A,B)** Percentage of CD45RA+CD45RO– **(A)** and CD45RA-CD45RO+ **(B)** cells within the CD4+ T cell population, and **(C,D)** percentage of CD45RA+CD45RO– **(C)** and CD45RA-CD45RO+ **(D)** cells within the CD8+ T cell population of ASM non-responders (NR) and responders (RES). **(E)** Percentage of CD3+CD56+NK T cells in 5TSTS NR and RES. **(F)** Naïve/memory cell ratio (CD45RA+CD45RO–/CD45RA-CD45RO+) in the CD8+ cell populations of ASM NR and RES. In panel **(F)**, significant differences between the ratio at baseline and week 12 in ASM RES (two-way ANOVA with *post hoc* Fisher’s LSD test) are indicated under the bar. Significance of difference between ASM NR and RES (two-way ANOVA with *post hoc* Fisher’s LSD test) is indicated above the error bar of ASM NR. NR, non-responder; RES, responder; ASM, appendicular skeletal muscle mass; 5TSTS, five times sit-to-stand test; ns, not significant. Unpaired *t*-test for panels **(A–E)**. *P < 0.05. Data are presented as the mean ± SEM.

**FIGURE 4:**
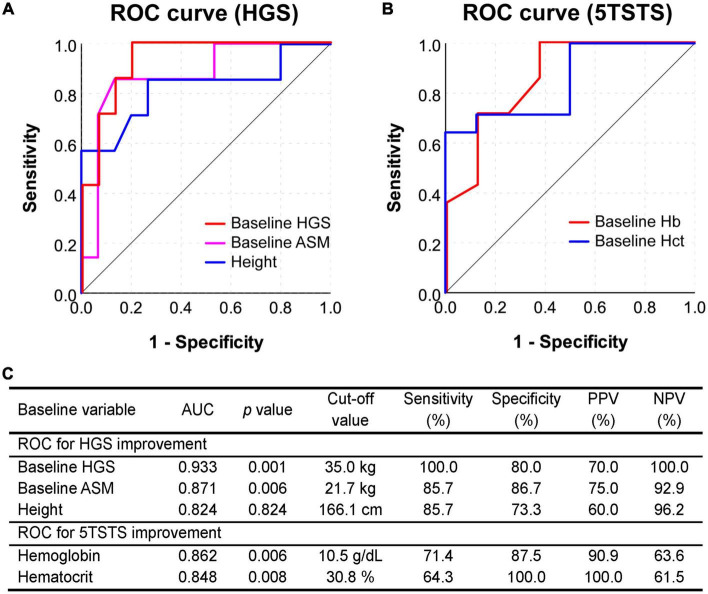
Receiver operating characteristic (ROC) curve analysis to assess the predictive value of baseline parameters. **(A)** ROC curve for predicting the HGS improvement (5% or more) based on the baseline HGS, ASM, and height. **(B)** ROC curve for predicting the 5TSTS improvement (5% or more) based on baseline Hb and Hct levels. ROC curves in this figure are as drawn smaller test result indicates more positive test. **(C)** The area under curve (AUC), cut-off values, sensitivity and specificity, and positive and negative predictive values of the test are presented in the table. HGS, handgrip strength; 5TSTS, five-times sit-to-stand test; ASM, appendicular skeletal muscle mass; Hb, hemoglobin; Hct, hematocrit.

In this pilot trial, we decided sample size based on previously published pilot trial sample size calculation, which recommended minimum of 20 patients in total (26, 27). Considering dropout, final sample size was set at 22. Group data are presented as the mean ± standard deviation unless noted otherwise. Unpaired *t*-tests were used to compare mean values between two groups (Figures 2, 3). Chi square tests were used for dichotomous variables (Table 2 and Supplementary Tables 2, 4). In comparisons of variables from three sequential time points, repeated measures ANOVA with a *post hoc* Fisher’s LSD tests were used (Supplementary Tables 3, 5, 7). Linear regression (Supplementary Table 6) and logistic regression analyses (Table 3) were used to identify key baseline variables that affect muscle strength and physical performance. When analyzing Receiver Operator Characteristics (ROC) curves, the cut-off value was determined using the Youden index method (Figure 4). Correlations between parameters were analyzed using Pearson’s correlation analysis (Supplementary Figure 4). Statistical significance for all analyses was established at a two-tailed *p* value of <0.05. Statistical analysis and graphing were performed using SPSS Version 26.0. (IBM corp., Armonk, NY, USA), GraphPad Prism 9.4.1. (GraphPad Software Inc., San Diego, CA, USA), and OriginPro 2021 (OriginLab corp., Northampton, MA, USA). Graphical abstract in Supplementary Figure 5 is created with BioRender.com.

## Results

### Baseline characteristics of the study participants

In this single center, prospective, single arm pilot study, a total of 22 patients were enrolled according to the eligibility criteria. The age of the patients ranged from 27 to 72 years with an average of 55.3 years, and the mean dialysis vintage was 4.7 years (Table 1). All the patients had been undergoing regular HD using high flux dialyzers. Among the cohort, seven out of 22 were female, accounting for 31.8% of total subjects. At baseline, the dialysis adequacy marker Kt/V was 1.51 ± 0.37, and 20 participants (90.9%) met the minimum target of Kt/V ≥ 1.2 for HD patients (28). Body composition analysis showed that none of the participants had low appendicular muscle mass according to the 2019 AWGS criteria (<7.0 ASM/m^2^ in males or <5.7 ASM/m^2^ in females). In the HGS test, only one female participant had low muscle strength (HGS < 18 kg for females). Based on the 2019 AWGS diagnostic criteria, seven cases of low physical performance were identified from the SPPB at baseline, including four cases of gait speed less than 1.0 m/s and three cases of sit-to-stand time with 12s or more. Among them, one participant showed both slow gait speed and delayed sit-to-stand time. Nonetheless, the SPPB scores of all participants were greater than nine (3). Taken together, at the start of the study, all participants were not sarcopenic (Table 1).

**TABLE 1:** Baseline characteristics of participants.

Characteristics	All participants (*N* = 22)
Age (year)	55.3 ± 11.8
Female, *n* (%)	7 (31.8%)
Height (cm)	166.4 ± 10.4
Post-HD Bw (kg)	66.2 ± 11.4
Post-HD BMI (kg/m^2^)	23.8 ± 2.9
HD duration (years)	4.7 ± 3.5
Kt/V	1.51 ± 0.37
Kt/V ≥ 1.2	20 (90.9%)
Causes of ESRD, *n* (%)	
DM	5 (22.7%)
GN	5 (22.7%)
HTN	4 (18.2%)
PCKD	3 (13.6%)
Cholesterol emboli	1 (4.5%)
Not known	4 (18.2%)
Fat mass (kg)	20.3 ± 7.8
Appendicular skeletal muscle mass (kg)	25.3 ± 6.9
Skeletal muscle index (ASM/m^2^)	9.0 ± 0.4
Low appendicular muscle mass	
Male (<7.0 ASM/m^2^, *n*)	0
Female (<5.7 ASM/m^2^, *n*)	0
Handgrip strength (kg)	32.8 ± 9.9
Low muscle strength	
Male (<28 kg, *n*)	0
Female (<18 kg, *n*)	1 (14.3%)
Low physical performance	5 (22.7%)
Gait speed (<1.0 m/s)	4 (18.2%)
Five times sit-to-stand test (≥12 s)	3 (13.6%)
SPPB (≤9 point)	0
Compliance for exercise (%)	87.1 ± 15.3
Compliance for leucine supplementation (%)	75.8 ± 22.3
Leucine side effects	10 (45.5%)
Dyspepsia	4 (18.2%)
Bloating	2 (9.1%)
Diarrhea	5 (22.7%)

HD, hemodialysis; Bw, body weight; BMI, body mass index; ESRD, end-stage renal disease; DM, diabetes mellitus; GN, glomerulonephritis; HTN, hypertension; PCKD, polycystic kidney disease; ASM, appendicular skeletal muscle mass; SPPB, short physical performance battery. Data are presented as the mean ± SD.

**TABLE 2:** Summary of responses of each individual in terms of the skeletal muscle index, HGS, and physical performance tests after the first 12 weeks of active supplementation and the second 12 weeks of observation.

		SMI		HGS		Gait speed		5TSTS	
**Number**	**Sex**	**0–12 week**	**12–24 week**	**0–12 week**	**12–24 week**	**0–12 week**	**12–24 week**	**0–12 week**	**12–24 week**
1	F	RES	–	RES	–	RES	–	–	–
2	F	RES	–	RES	–	–	RES	RES	–
3	F	RES	–	RES	–	–	RES	RES	–
4	F	RES	RES	RES	–	–	RES	–	RES
5	F	RES	RES	–	–	RES	–	RES	–
6	F	RES	–	–	–	–	RES	RES	–
7	F	–	RES	RES	–	–	–	RES	–
8	M	RES	–	RES	–	–	RES	RES	RES
9	M	RES	–	–	–	RES	RES	RES	–
10	M	RES	RES	–	–	RES	–	RES	RES
11	M	RES	–	–	–	RES	–	RES	RES
12	M	RES	–	–	–	RES	–	RES	–
13	M	RES	RES	–	–	RES	–	–	–
14	M	RES	–	–	RES	RES	–	–	RES
15	M	RES	RES	–	–	–	–	–	RES
16	M	–	RES	RES	–	RES	–	–	RES
17	M	–	RES	–	–	RES	RES	RES	–
18	M	–	–	–	–	RES	–	RES	–
19	M	–	RES	–	–	RES	–	RES	–
20	M	–	–	–	–	RES	RES	–	RES
21	M	–	RES	–	–	–	RES	RES	–
22	M	–	–	–	–	–	RES	–	–
An error in the conversion from LaTeX to XML has occurred here. 2*Responder N (%)		14	10	7	1	13	10	14	8
		(63.6%)	(45.5%)	(31.8%)	(4.5%)	(59.1%)	(45.5%)	(63.6%)	(36.4%)
*p*-value		0.113		0.001**		0.183		0.035*	

RES, responsive individual; SMI, skeletal muscle index; HGS, handgrip strength; 5TSTS, five times sit-to-stand test. Chi-square test for statistical analysis. *p < 0.05, **p < 0.01.

**TABLE 3:** Association between HGS and 5TSTS responsiveness and baseline clinical variables.

Parameter	Baseline variable	Univariate analysis		Multivariate analysis	
		Odds ratio	*p*-value	Odds ratio	*p*-value
HGS	Sex (ref. male)	0.062	0.014	0.780	0.010
	Exercise compliance	16.500	0.022		
	Baseline grip strength	0.784	0.011		
	ASM	0.711	0.016		
	Height	0.862	0.028		
	Total cholesterol	1.044	0.045		
5TSTS	RBC	0.010	0.022	0.524	0.025
	Hemoglobin	0.130	0.011		
	Hematocrit	0.520	0.016		
	CD8+CD45RA+CD45RO+	5.60E + 09	0.028		
	CD8+CD45RO+CD279+	0.180	0.045		

HGS, handgrip strength; 5TSTS, five times sit-to-stand test; ASM, appendicular skeletal muscle mass.

### Nutritional status before and after 12 weeks of intervention

To cope with the loss of energy and protein caused by dialysis, guidelines recommend that CKD patients should intake an appropriate amount of energy and protein (29, 30). The recommended energy intake for CKD patients, with or without dialysis, is 25–35 kcal/kg of body weight per day, and the recommended protein intake for CKD patients receiving maintenance HD is 1.0–1.2 g/kg of body weight per day (29, 30). At the beginning, 12 week, and 24 weeks of the study, we evaluated the nutritional status of the patients using the 24-hour dietary recall method. We found that, at baseline, only four (18.2%) and three (13.6%) patients met the energy and protein intake recommendations, respectively (Supplementary Table 2). After 12 weeks of leucine-enriched supplementation and resistance exercise, the amount of energy and protein intake significantly increased (from 991.6 ± 387.5 to 1219.4 ± 249.5 kcal, *p* < 0.001; from 33.3 ± 15.7 to 48.7 ± 9.6 g of protein, *p* < 0.001). Although the number of patients who met the energy and protein intake recommendations did not increase, the proportion of patients consuming less than half of the recommendations significantly decreased (Supplementary Table 2). In terms of energy intake, the number of patients consuming less than half of the recommended energy intake (<12 kcal/kg/day) significantly fell from 9 (40.9%) to none (*p* = 0.001). In terms of protein intake, the number of patients consuming less than half of the recommended protein intake (<0.6 g/kg/day) also significantly fell from 13 (59.1%) to only one patient (4.5%) (*p* < 0.001). The leucine-enriched amino acid beverage contains not only amino acids but also carbohydrates, lipids, vitamins, and minerals (Supplementary Table 1). Therefore, the amount of nutrients taken during the intervention significantly increased not only in macronutrients but also in micronutrients, including vitamin D (from 0.7 ± 1.5 to 23.2 ± 7.6 μg, *p* < 0.001) and calcium (from 200.7 ± 118.7 to 816.5 ± 231.2 mg, *p* < 0.001) (Supplementary Table 2). Importantly, leucine intake was increased from 1.6 ± 0.9 to 5.8 ± 1.8 g (*p* < 0.001). These data suggest that leucine-enriched amino acid supplementation effectively provided leucine to the patients and improved their intake of energy and protein, as well as micronutrients.

### Effects of leucine-enriched amino acid supplementation and resistance exercise on muscle mass and function

Participants received leucine-enriched amino acid supplementation and exercised daily following exercise program for the first 12 weeks, as “interventional period”. For the next 12 weeks, the supplements were not provided and patients did not exercise regularly, as “non-interventional period” (Figure 1). The response of each participant during the interventional and non-interventional period is shown in Table 2. We classified the participants into responders and non-responders based on percent change in the parameters. The patients whose change in HGS and physical performance tests (including gait speed and 5TSTS) improved more than 5% compared to the previous time point was defined as responders (RES). In skeletal muscle index (SMI), participants who did not lose skeletal muscle mass during the 12-week interval were regarded as responders (RES). The response rate was defined as the proportion of RES individuals among the total 22 participants during the first and second 12 weeks of interval. After the leucine-enriched amino acid and exercise intervention, SMI and 5TSTS were improved in 14 patients (63.6%), respectively. HGS was improved in 7 patients (31.8%), and gait speed was improved in 13 patients (59.1%). During the interventional period, only one patient (Number 22 in Table 2) did not show any improvement, and other 21 patients (95.4%) showed improvement in at least one parameter among SMI, HGS, gait speed, and 5TSTS. Interestingly, during the non-interventional period, the response rates of SMI and gait speed were not significantly decreased compared to interventional period (SMI from 63.6 to 45.5%, *p* = 0.113; gait speed from 59.1 to 45.5%, *p* = 0.183). However, the response rates of HGS and 5TSTS were significantly decreased during non-interventional period compared to the interventional period (HGS from 31.8 to 4.5%, *p* = 0.001; 5TSTS from 63.6 to 36.4%, *p* = 0.035). This suggests that the positive effects of the leucine-enriched amino acid and exercise intervention on HGS and 5TSTS were not sustained during the non-interventional period (Table 2). In average of total patients in each time point, gait speed was the only parameter that showed improvement during the non-interventional period, with the average gait speed increased from 1.35 ± 0.20 to 1.41 ± 0.21 m/s (*p* = 0.04) (Supplementary Table 3). In contrast, although 5TSTS showed improvement during the interventional period (from 7.8 ± 2.9 to 6.5 ± 1.9 s, *p* = 0.016), they did not change significantly during the non-interventional period on average (from 6.5 ± 1.9 to 6.2 ± 1.8 s, *p* = 0.262) (Supplementary Table 3). The average HGS remained unchanged during the first 12 weeks (32.8 ± 9.9 to 32.8 ± 8.8 kg, *p* = 0.915) but decreased during the second 12 weeks (from 32.8 ± 8.8 to 32.0 ± 9.4 kg, *p* = 0.060), reflecting the low HGS response rate during non-interventional period (4.5%) (Table 2 and Supplementary Table 3). Regarding body composition, while SMI did not significantly change over 24 weeks (from 9.2 ± 2.5 to 9.2 ± 2.3 kg/m^2^, *p* = 0.851), body weight and BMI decreased during the non-interventional period (Body weight from 66.5 ± 11.5 to 65.4 ± 11.0 kg, *p* = 0.009; BMI from 23.9 ± 3.1 to 23.7 ± 3.1 kg/m^2^, p = 0.031) (Supplementary Table 3 and Supplementary Figures 1A–C). The body weight loss observed across the total study duration could be explained by reduction in fat mass and total body water for 24 weeks (fat mass from 20.3 ± 7.8 to 19.2 ± 7.6 kg, *p* = 0.046; total body water from 34.8 ± 8.0 to 30.5 ± 6.9 L, p < 0.001). During the non-interventional period, intracellular water loss was more prominent than extracellular water loss (ECW/ICW ratio from 0.8 ± 0.1 to 0.9 ± 0.1, *p* = 0.007) (Supplementary Figures 1D–I). These data suggest that the intervention improved muscle mass and physical function, but the positive effects on HGS and 5TSTS were not sustained during the non-interventional period, while SMI and gait speed remained improved. And during the 24 weeks of study, fat mass and total body water were decreased, but ASM was not changed.

### Effects of the intervention on serum biochemistry

The results of serum chemistry tests reflected changes due to the high protein diet. Serum BUN increased from baseline to 12 week (from 60.9 ± 16.2 to 73.9 ± 16.3 mg/dl, *p* = 0.002), and potassium also increased during first 12 weeks (from 4.8 ± 0.7 to 5.2 ± 0.6 mEq/L, *p* = 0.041). Despite the elevation of potassium level, there were no major adverse cardiac events. Although the non-interventional periods led to decreases in elevated BUN and potassium levels, the changes were not statistically significant (BUN from 73.9 ± 16.3 to 66.9 ± 14.0 mg/dl, *p* = 0.100; potassium from 5.2 ± 0.6 to 4.9 ± 0.7 mEq/L, *p* = 0.195) (Supplementary Table 3). During the interventional period, there was no increase in average total protein, albumin, phosphorus, and uric acid levels (total protein from 6.6 ± 0.4 to 6.7 ± 0.4 g/dl, *p* = 0.356; albumin from 3.7 ± 0.3 to 3.8 ± 0.3 g/dl, *p* = 0.144; phosphorus from 4.9 ± 1.0 to 5.0 ± 1.5 mg/dl, *p* = 0.799; uric acid from 7.2 ± 1.4 to 7.5 ± 1.2 mg/dl, *p* = 0.310) (Supplementary Table 3). Serum biochemistry also reflected the effects of exercise. HDL cholesterol increased after the first 12 weeks (from 41.2 ± 12.3 to 44.6 ± 12.7 mg/dl, *p* = 0.045), and the monocyte fraction decreased after 24 weeks (from 7.4 ± 1.6 to 6.8 ± 1.5%, *p* = 0.026), supporting the effects of exercise on monocyte numbers (31). Collectively, The intervention, which included a high protein diet and resistance exercise, resulted in increases in BUN and potassium levels, as well as an increase in HDL cholesterol, without causing major adverse cardiac events for 24 weeks.

### Baseline factors that predict improvements in muscle mass and function after leucine-enriched amino acid supplementation and resistance exercise

To identify baseline factors that could predict improvement in muscle mass, strength, and physical performance after 12 weeks of intervention, we first compared baseline variables between non-responder (NR) and responder (RES) groups. In ASM RES, the baseline monocyte fraction was lower than in ASM NR (ASM NR versus RES; 8.4 ± 1.9 versus 6.9 ± 1.1%, *p* = 0.029) (Figure 2A). The ASM RES tended to lose fat mass during the first 12 weeks of intervention (ASM NR versus RES; 1.2 ± 1.7 versus −1.1 ± 1.7%, *p* = 0.002) (Supplementary Figure 2A). In HGS RES, baseline serum total protein was lower, and change in total protein was higher, than in HGS NR (HGS NR versus RES; total protein, 6.7 ± 0.4 versus 6.4 ± 0.3 g/dl, *p* = 0.036; change in total protein, 0.0 ± 0.4 versus 0.3 ± 0.2 g/dl, *p* = 0.016) (Figure 2B and Supplementary Figure 2B). In 5TSTS RES, baseline hemoglobin (Hb) was lower, and changes in Hb and serum sodium were higher, than in 5TSTS NR (5TSTS NR versus RES; Hb, 11.2 ± 0.9 versus 9.8 ± 1.0 g/dl, *p* = 0.004; change in Hb, −0.8 ± 1.5 versus 0.7 ± 1.2 g/dl, *p* = 0.019; change in Na, −2.9 ± 2.7 versus 0.1 ± 2.9 mEq/L, *p* = 0.039 (Figure 2C and Supplementary Figures 2C, D). Comparison of demographic factors revealed that females gained more HGS than males during interventional periods (female versus male; 7.6 ± 8.2 versus −1.6 ± 7.2%, *p* = 0.026) (Figure 2D). In comparisons of muscle mass, strength, and physical performances between female and male patients, female showed lower baseline HGS but higher response rate (female versus male; HGS, 21.2 ± 2.9 versus 38.2 ± 6.6 kg, *p* < 0.001; HGS response rate, 71.4 versus 13.3%, *p* = 0.014) as demonstrated in Figure 2D and Table 2 (Supplementary Table 4). In addition, females had a lower body surface area than males (female versus male; 1.53 ± 0.11 versus 1.85 ± 0.13 m^2^, *p* < 0.001) without significant difference in the amount of leucine intake (female versus male; 3.9 ± 2.0 versus 4.3 ± 1.8 g, *p* = 0.585). However, leucine uptake per BSA was not significantly different between female and male (female versus male; 2.5 ± 1.3 versus 2.4 ± 1.0 g/m^2^, *p* = 0.779) (Supplementary Table 4). In addition, patients over age of 60 showed more significant gain in HGS than those age under 60 (age > 60 versus ≤ 60; 5.3 ± 6.2 versus –1.4 ± 9.1%, *p* = 0.043) (Figure 2E). Participants whose exercise compliance was ≥ 95% showed greater improvements in HGS and serum albumin levels (exercise compliance ≥ 95% versus < 95%; change in HGS, 6.8 ± 7.7 versus –3.2 ± 6.4%, *p* = 0.004; change in albumin, 0.0 ± 0.2 versus 0.2 ± 0.2 g/dL, *p* = 0.047) (Figure 2F and Supplementary Figure 2E). These data provide baseline factors of RES as low monocyte fraction in ASM RES, low total protein in HGS RES, low Hb in 5TSTS RES. And, in HGS, old age (>60), female, high (≥95%) exercise compliance had more improvement.

### Immunophenotypic characteristics of responders of muscle mass and physical performance

Immunophenotyping of participants’ PBMC revealed additional features in RES. ASM responders (RES) had lower naïve CD4+ T cell (CD4+CD45RA+CD45RO-) fraction and higher CD4+ memory T cell (CD4+CD45RA-CD45RO+) fraction at baseline than NR (ASM NR versus RES; CD4+ naïve, 53.2 ± 7.5 versus 37.1 ± 15.5%, *p* = 0.013; CD4+ memory, 38.2 ± 7.5 versus 53.8 ± 15.2%, *p* = 0.014) (Figures 3A, B). CD8+ T cell in ASM RES showed the same trend but the difference in naïve CD8+ T cell fraction was not statistically significant (ASM NR versus RES; CD8+ naïve, 52.4 ± 16.7 versus 41.6 ± 14.5%, *p* = 0.095; CD8+ memory, 30.4 ± 11.9 versus 42.7 ± 13.6%, *p* = 0.045) (Figures 3C, D). During the first 12 weeks of the intervention, the ratio of naïve/memory T cells (CD45RA+CD45RO-/CD45RA-CD45RO+) within the CD4+ T cell population did not change significantly both in ASM NR and ASM RES (ASM NR from 1.5 ± 0.5 to 1.5 ± 0.5, *p* = 0.747; ASM RES from 0.8 ± 0.6 to 0.7 ± 0.5, *p* = 0.442) (Supplementary Figure 3A), whereas the ratio of naïve/memory CD8+ T cell tended to increase in ASM RES (ASM RES from 1.2 ± 0.8 to 1.4 ± 1.1, *p* = 0.073) (Figure 3F). In addition, 5TSTS RES had higher baseline CD3+CD56+NK T cell fraction than NR (5TSRS NR versus RES; 0.2 ± 0.2 versus 2.3 ± 2.5, *p* = 0.045) (Figure 3E). In contrast, subjects who experienced fat mass reduction (fat mass RES) had lower baseline CD3+CD56+NK T cell numbers and higher CD3+CD56- cell numbers than fat mass NR (fat mass reduction NR versus RES; CD3+CD56+, 2.2 ± 2.5 versus 0.2 ± 0.2%, *p* = 0.026; CD3+CD56-, 93.5 ± 5.4 versus 99.6 ± 0.4%, *p* = 0.014) (Supplementary Figures 3B, C). In fat mass reduction RES, the baseline CD14+CD16- cell fraction which is a classical monocyte was lower than that of NR (fat mass reduction NR versus RES; 73.4 ± 12.2 versus 57.1 ± 19.4%, *p* = 0.028) (Supplementary Figure 3D). The effects of the diet and exercise intervention on the immunophenotype of PBMCs are shown in Supplementary Table 5. CD3+CD56+NK T cell fraction fell during the first 12 weeks (from 1.7 ± 2.3 to 1.4 ± 2.0%, *p* = 0.010), and remained unchanged during the next 12 weeks (from 1.4 ± 2.0 to 1.4 ± 1.8%, *p* = 0.907). Although there was no significant increase in the fractions of CD45RA+ cells during the study, there were significant falls in CD45RO+ cells including CD4+CD45RO+CD279+ (for 24 weeks, from 1.9 ± 3.4 to 0.8 ± 1.2%, *p* = 0.051), CD8+CD45RO+CD197+ (for 24 weeks, from 2.0 ± 1.1 to 1.3 ± 1.0%, *p* = 0.004), and CD8+CD45RO+CD279+ cells (for 24 weeks, from 1.9 ± 1.6 to 1.1 ± 1.2%, *p* = 0.032). Immune cells expressing TNFα (CD4+CD197+TNFα+; for 24 weeks, from 2.0 ± 1.5 to 1.2 ± 1.2%, *p* = 0.010) and IL17A (CD4 + CD197 + IL17A +; for 24 weeks, from 0.7 ± 0.9 to 0.2 ± 0.2%, *p* = 0.015) were significantly decreased (Supplementary Table 5). Taken together, immunophenotyping of patients’ PBMC revealed that ASM RES had lower naïve T cells at baseline than ASM NR, and during the 24 weeks of study, CD45RO+ cells as well as immune cells expressing TNFα and IL17A were decreased.

### Baseline factors for prediction of HGS and 5TSTS improvement and their validation using ROC curves

To identify factors showing a linear relationship with improvements in muscle mass, strength, or physical function, we performed multivariate linear regression analysis (Supplementary Table 6). Change in HGS was related to a low baseline HGS, high exercise compliance, and low baseline CD8+CD57+TNFα+ cells, a marker of immunosenescence, at baseline (baseline HGS, β = –0.534 and *p* = 0.002; exercise compliance, β = 10.042 and *p* = 0.003; baseline CD8+CD57+TNFα+, β = –2.270 and *p* = 0.027). Change in gait speed was associated with slow baseline gait speed and low mean corpuscular hemoglobin concentration (MCHC) at baseline (baseline gait speed, β = –12.584 and *p* < 0.001; baseline MCHC, β = –16.216 and *p* = 0.005). Finally, Change in 5TSTS was associated with low baseline 5TSTS and high CD8+CD45RO+CD279+ cells at baseline, which are the exhausted and dysfunctional CD8+ T cells suggestive of a chronic inflammatory status (baseline 5TSTS, β = –5.453 and *p* = 0.002; baseline CD8+CD45RO+CD279+, β = 10.908 and *p* = 0.018) (Supplementary Table 6). The correlation plot summarizes the relationship between factors affecting muscle mass, strength, and physical functions (Supplementary Figure 4). Change in HGS, gait speed, and 5TSTS was negatively correlated with baseline HGS, gait speed, and 5TSTS, respectively (HGS, *r* = –0.615 and *p* = 0.002; gait speed, *r* = –0.847 and *p* < 0.001; and 5TSTS, *r* = –0.613 and *p* = 0.002). Baseline HGS correlated positively with baseline ASM (*r* = 0.906 and *p* < 0.001), while change in HGS correlated negatively with baseline ASM (*r* = –0.600 and *p* < 0.003). The baseline monocyte fraction correlated negatively with baseline gait speed (*r* = –0.489 and *p* = 0.021), but positively correlated with change in gait speed (*r* = 0.491 and *p* = 0.020) (Supplementary Figure 4). In addition, change in fat mass was negatively correlated with change in ASM (*r* = –0.582 and *p* = 0.004). Using binary logistic regression analysis, we found predictive factors of HGS and 5TSTS improvement (Table 3). A low baseline HGS was the factor most strongly associated with HGS improvement (OR = 0.780, *p* = 0.010), and low baseline hematocrit was the factor most strongly associated with 5TSTS improvement (OR = 0.524, *p* = 0.025).

Validation of the factors derived from regression analyses was performed by plotting the ROC curve and calculating the area under the curve (AUC). In ROC curve of Figure 4A, smaller test results of baseline HGS, ASM, and height indicate more positive test and, in ROC curve of Figure 4B, smaller test results of hemoglobin and hematocrit indicate more positive test. A low HGS at baseline showed the largest AUC (0.933) for predicting an HGS response, with a cut-off value of 35.0 kg (Figure 4A). The baseline HGS test had sensitivity of 100.0%, specificity of 80.0%, positive predictive value (PPV) of 70.0%, and negative predictive value (NPV) of 100.0% for the prediction of HGS improvement (Figure 4C). On the other hand, low baseline low Hb showed the largest AUC (0.862) for predicting a 5TSTS response, with a cut-off value of 10.5 g/dl (Figure 4B). The baseline Hb test had sensitivity of 71.4%, specificity of 87.5%, PPV of 90.9%, and NPV of 63.6% for the prediction of 5TSTS improvement (Figure 4C). Taken together, we identified baseline factors associated with improvements in HGS and 5TSTS, and found that baseline HGS less than 35.0 kg and baseline Hb less than 10.5 g/dL are the strongest factor that predict HGS and 5TSTS improvements, respectively.

### Adverse events

During the intervention period, compliance to exercise (87.1 ± 15.3%) and leucine supplementation (75.8 ± 22.3%) was monitored weekly (Table 1). Ten participants (45.5%) complained of leucine supplementation-related side effects such as dyspepsia (*N* = 4, 18.2%), bloating (*N* = 2, 9.1%), and diarrhea (*N* = 5, 22.7%). In three of these participants, symptoms were self-limited, so they continued supplementation; however, seven of them discontinued the leucine capsules and reduced their leucine-enriched beverage intake by one-to-two thirds. Regardless of the discontinuation of leucine capsules by seven participants, leucine supplementation resulted in an additional leucine intake of 4.2 g in average compared to baseline (from 1.6 ± 0.9 to 5.8 ± 1.8 g, *p* < 0.001) (Supplementary Table 2). No grade 2 or higher adverse events, based on WHO toxicity grading system, were reported during the 24-week study period. These data suggest that side effects of the intervention were self-limited and do not significantly affect the overall compliance to exercise and leucine supplementation.

## Discussion

This single center, prospective single-arm interventional pilot trial investigated changes in muscle mass, strength, physical function, serum chemistry, and the immunophenotype of PBMCs during 12 weeks of a leucine-enriched amino acid supplementation and resistance exercise intervention, and followed by another 12 weeks of non-intervention period. Overall, 95.4% of patients showed improvement in at least one parameter among muscle mass, strength, and physical functions (63.6% in SMI, 31.8% in HGS, 59.1% in gait speed, and 63.6% in 5TSTS) during the first 12 weeks of interventional period. The improvements in SMI and gait speed were maintained for up to 24 weeks, whereas those in HGS and 5TSTS fell significantly during the second 12 weeks of non-interventional period (Table 2). In addition, we analyzed the baseline characteristics of the subjects in whom the intervention resulted in improvements in muscle mass, strength, or physical performance. Subjects with a baseline HGS less than 35.0 kg are expected to improve their HGS, and subjects with a baseline hemoglobin less than 10.5 (g/dl) are expected to improve their 5TSTS (Figure 4). Immunophenotype analysis found that CD8+ naïve/memory T cell fraction was increased after the intervention. In brief, we evaluated the effects of leucine-enriched amino acid supplementation and resistance exercise and identified the baseline characteristics of non-sarcopenic HD patients likely to show improvement in muscle mass, strength, and physical performance after the intervention (Supplementary Figure 5 for graphical abstract).

In HD patients, multiple factors promote a negative protein balance resulting in sarcopenia. Uremia-induced poor oral intake is one reason for the imbalance of protein homeostasis. In our cohort, only 13.6% of subjects had protein intake more than 1.0 g/kg/day, and 59.1% of subjects consumed less than 0.6 g/kg/day at baseline. Real-world data from HD patients in European countries show that 67% have a dietary protein intake more than 1.1 g/kg/day (32, 33). Current KDOQI clinical practice guidelines for nutrition in CKD recommend 1.0–1.2 g/kg/day protein intake for maintenance HD patients (29). Though leucine-enriched amino acid supplementation did not increase the number of subjects meeting the recommended protein intake, the proportion of taking less than 0.6 g/kg/day fell significantly (Supplementary Table 2). Loss of amino acids during HD is also an important reason for the imbalance of protein homeostasis. During a 4 h HD session, patients lose 6–12 g amino acids into dialysate by using high-flux dialyzer (13). As a result, though the basal muscle protein synthesis in MHD patients is ∼2-fold higher than in controls, MHD patients fail to upregulate post-prandial muscle protein synthesis, which condition is termed as anabolic resistance (20). In addition to protein loss, low baseline intake of vitamin D in our cohort which was 0.7 μg per day could be another nutritional risk factor for sarcopenia.

Several other factors could promote protein degradation in HD patients, leading to catabolic status. Chronic low-grade inflammation originated from accumulated uremic toxins, gut dysbiosis, and dialysis membrane biocompatibility could promote protein degradation (34–36). Previous studies reported that reduced fraction of CD45RA+ naïve T cells and increased fraction of immunosenescent CD57+ T cells as well as proinflammatory CD4+CD28- memory T cells, and CD14+CD16++ monocytes are hallmarks of inflammaging in ESRD patients (37, 38). In the current study, ASM responders had low CD45RA+ naïve T cell and high CD45RO+ memory T cell fraction at baseline, and their CD8+ naïve/memory T cells were increased after the intervention (Figures 3, Supplementary Figure 3A, and Supplementary Table 7). This means that even if participants are on a status of inflammaging at baseline, they could still gain in ASM through the intervention. Improvement of inflammation status is also supported by reduction of TNFα+ or IL17A+ cell fraction in our cohort (Supplementary Table 5). Our data suggest that leucine-enriched amino acid supplementation and resistance exercise reduce inflammatory responses. Elaborately structured RCTs investigating markers of inflammaging and cytokine levels in ESRD are needed to solidify these findings. Finally, previous studies demonstrate the role of metabolic acidosis in increased protein catabolism, which includes reducing circulating leucine and albumin synthesis (39, 40). Although the results were not significant, total CO_2_ increased gradually during the 24 weeks (from 19.1 ± 3.6 at baseline to 20.5 ± 1.9 at Week 24, *p* = 0.054). Collectively, results from this study suggest that leucine-enriched amino acid supplementation and resistance exercise protect against multiple factors that promote a negative protein balance. Furthermore, baseline characteristics of lower total protein in HGS responders, lower hemoglobin and hematocrit in 5TSTS responders, and a lower naïve/memory T cell in ASM responders compared to each non-responder group suggest that responders have more baseline potential for improvement by the intervention.

In immunophenotype analysis we found that 5TSTS responders had higher baseline CD3+CD56+NK T cell subset which is known to be elevated in obese individuals (41), and the responders had higher BMI than non-responsers (24.8 ± 2.6 in responders versus 22.3 ± 3.5 kg/m^2^ in non-responders, *p* = 0.037). In comparison of baseline CD3+CD56+ cells between fat mass reduction responders and non-responder, interestingly, responders who had lose 5% or more fat mass had lower CD3+CD56+ fraction than non-responders. Taken together, in our cohort, patients who have baseline high CD3+CD56+ fraction showed improvement in 5TSTS, but they did not lose fat mass. Baseline CD14+CD16- cell fraction, which is a classical monotye and a predictor of cardiovascular events (42), was lower in fat mass reduction responders than non-responder, and it could be explainable that baseline BMI was positively correlated with change in fat mass (*r* = 0.447, *p* = 0.037; Supplementary Figure 4) and it means fat mass reduction responder would have low baseline BMI.

There have been several previous studies investigating the effect of amino acid/protein supplementation in patients undergoing HD. These studies provided supplements on dialysis day, with either 30 g of whey or 27 g of soy protein, containing only 2.7 and 2.3 g of leucine, respectively. Furthermore, their outcome measures focused only on components of the sarcopenia diagnostic criteria. In a meta-analysis summarizing these studies, amino acid/protein supplementation failed to demonstrate improvement in muscle mass and strength, but did show significant improvement in physical function, including shuttle walking, gait speed, and timed up and go (43). Recent approach of the sarcopenia prevention included not only protein supplementation but also resistance exercise to attenuate anabolic resistance of HD patients which is originated from a low physical activities (44). However, to date, only a few RCTs have examined a combined diet and exercise intervention for HD patients. One RCT evaluated the effects of intradialytic whey protein intake (30 g) and endurance exercise (up to 45 min on a cycle ergometer) on physical function, arterial stiffness, blood pressure, body composition, muscle strength, nutritional status, and quality of life (45). Unfortunately, they cannot demonstrate significant improvements in physical function, risk of cardiovascular disease, or quality of life over 1 year. Compared to our cohort, the study population had a higher BMI (31.4 ± 7.7 kg/m^2^), a greater prevalence of DM (47.8%), and a lower leucine intake (9 g/week), which may have result in these findings. Another RCT evaluated oral nutritional supplementation with or without exercise. They provided two cans of specialized oral nutritional supplement, which contains 434 kcal, 19.2 g protein without leucine, and 22.8 g lipids per a can, for dialysis patients on the day of dialysis. They found that, although muscle mass was not improved, oral nutrition plus exercise group had greater improvement in 6-min walk test and timed up and go test over oral nutrition alone group. To our knowledge, the current study is the first trial of combined leucine-enriched amino acid supplementation and resistance exercise for HD patients. Large-scale studies are needed to strengthen the evidence of protein supplementation and exercise in HD patients and to overcome the limitations of previous studies.

The strengths of this study are as follows: first, patients’ responses to the intervention were investigated in a multifaceted manner. Muscle-related clinical parameters as well as serum biochemistry and immunophenotyping of PBMCs were examined in this study. Second, we identified baseline characteristics of patients whose HGS and 5TSTS improved. The novelty of this study lies in this finding and we believe that it will help to select patients for protein supplementation and exercise in the clinical field. Third, we determined the nutritional status of the subjects using 24 h recall method and dedicated software to calculate the amount of macro- and micro- nutrients consumed. Compliance to the supplementation was monitored weekly.

The study has several limitations. First, this study is a single-arm trial. Because there is no simultaneous non-intervention control group, it is difficult to ascertain the direct effects of the intervention. Instead, we designed the study to include not only the interventional period but also a consecutive non-interventional period to compare two time points within the same group. This is not perfect because the effect during the intervention period may affect the subsequent non-intervention period; thus, a large-scaled randomized controlled trial for HD patients is required. Second, this is a pilot trial with small number (*N* = 22) of subjects with a single ethnicity and relatively short intervention period. Third, responders of skeletal muscle mass were defined as any increase in muscle mass due to small number (*N* = 3) of patients who showed 5% or more increase in ASM. Fourth, using bioimpedance methods to measure body composition of HD patients may lead to variable results due to the difference in volume status pre- and post-HD. To minimize this issue, we evaluated the body composition immediately after the HD session (46). Finally, the leucine-enriched beverage we gave to the patients included 400 IU of vitamin D and 290 mg of calcium, which can potentially aid in sarcopenia prevention (47, 48).

In summary, in this single center, prospective single arm, pilot trial, patients were given leucin-enriched amino acid supplements contain 6 g of leucine per day and performed daily resistance exercise. After 12 weeks of the intervention period, we observed improvements in SMI, HGS, gait speed, and 5TSTS, and after 12 weeks of non-interventional period, HGS and 5TSTS response rates were not maintained. Baseline low grip strength and low hematocrit were strong predictors for HGS and 5TSTS improvement, respectively. Moreover, old age female with good exercise compliance was the most benefited subgroup by this intervention in terms of HGS improvement. In immunophenotype analysis, naïve immune cell fraction was increased after the intervention. Some patients experienced grade 1 gastrointestinal symptoms that were self-limited, and no severe adverse events occurred. We propose that leucine-enriched amino acid supplementation and exercise are a safe and effective way to prevent muscle mass loss, preserve muscle strength, and maintain physical function in HD patients. Further studies are needed to evaluate the long-term effects of the intervention on muscle mass, strength, and physical performance.

## Data availability statement

The raw data supporting the conclusions of this article will be made available by the authors, without undue reservation.

## Ethics statement

The studies involving human participants were reviewed and approved by Institutional Review Boards of Chungnam National University Hospital (CNUH-2020-10-019-005). The patients/participants provided their written informed consent to participate in this study.

## Author contributions

YRH and H-SY: conceptualization, supervision, funding acquisition, and writing—review and editing. S-HJ, EJL, BCS, HTN, HYL, and JT: data curation. S-HJ, DEC, and HP: formal analysis. KJC and EJL: investigation. EJL, YRH, and H-SY: project administration. S-HJ and BCS: visualization. S-HJ and EJL: writing—original draft. All authors contributed to the article and approved the submitted version.
