# Supplementary material for: Leucine-enriched amino acid supplementation and exercise to prevent sarcopenia in patients on hemodialysis: a single-arm pilot study
Source: Front Nutr. 2023 Apr 28;10:1069651. doi: 10.3389/fnut.2023.1069651 (PMC10176607; doi:10.3389/fnut.2023.1069651)
Supplement: Supplementary file 1 [file Table_1.docx]

Supplementary Material

**Supplementary Table 1. Nutritional composition of the study products (amount per 125 ml of beverage)**

| Component | Amount |
| --- | --- |
| Energy (kcal) | 105 |
| Protein (g) | 8 |
| Leucine (mg) | 1,000 |
| Carbohydrate (g) | 8.5 |
| Fat (g) | 4.6 |
| Vitamin D (㎍) | 10 |
| Calcium (mg) | 290 |
| Vitamin A (㎍ RE) | 250 |
| Vitamin B1 (mg) | 0.6 |
| Vitamin B2 (mg) | 0.45 |
| Niacin (mg NE) | 3.2 |
| Vitamin B6 (mg) | 1.0 |
| Vitamin C (mg) | 20 |
| Vitamin E (mg α-TE) | 4.6 |
| Folate (㎍) | 85 |
| Pantothenic acid (mg) | 1.4 |
| Iron (mg) | 6 |
| Zinc (mg) | 5 |
| Biotin (㎍) | 8 |

㎍ RE, micrograms of retinol equivalents; mg NE, milligrams of niacin equivalents; mg α-TE, milligrams of α-tocopherol equivalents.

**Supplementary Table 2. Daily nutrient intake per meal, with or without leucine-enriched supplementation**

| Nutrient | Daily intake | | *p* value |
| --- | --- | --- | --- |
|  | By meal  at baseline | By meal  plus intervention |  |
| Energy (kcal) | 991.6 ± 387.5 | 1219.4 ± 249.5 | <0.001*** |
| < 12 kcal/kg/day | 9 (40.9%) | 0 | 0.001 |
| ≥ 12 kcal/kg/day | 13 (59.1%) | 22 (100.0%) |  |
| Energy intake > 25 kcal/kg/day | 4 (18.2%) | 3 (13.6%) | 0.689 |
| Carbohydrate (g) | 157.9 ± 64.2 | 181.6 ± 49.2 | 0.001** |
| Lipid (g) | 22.4 ± 14.9 | 31.5 ± 11.2 | <0.001*** |
| Protein (g) | 33.3 ± 15.7 | 48.7 ± 9.6 | <0.001*** |
| < 0.6 g/kg/day | 13 (59.1%) | 1 (4.5%) | <0.001*** |
| ≥ 0.6 g/kg/day | 9 (40.9%) | 21 (95.5%) |  |
| Protein intake > 1.0 g/kg/day | 3 (13.6%) | 3 (13.6%) | 0.459 |
| Vitamin A (μg RAE) | 248.2 ± 234.4 | 733.9 ± 206.2 | <0.001*** |
| Vitamin D (μg) | 0.7 ± 1.5 | 23.2 ± 7.6 | <0.001*** |
| Vitamin E (mg) | 7.6 ± 5.8 | 7.9 ± 4.5 | 0.671 |
| Vitamin C (mg) | 35.5 ± 52.0 | 71.7 ± 18.4 | <0.001*** |
| Thiamine (mg) | 0.9 ± 0.6 | 2.1 ± 0.5 | <0.001*** |
| Riboflavin (mg) | 0.7 ± 0.5 | 1.6 ± 0.4 | <0.001*** |
| Niacin (mg) | 5.8 ± 4.2 | 5.2 ± 2.4 | 0.319 |
| VitB6 (mg) | 0.7 ± 0.4 | 3.3 ± 1.3 | <0.001*** |
| Folate (μg) | 231.1 ± 222.0 | 397.9 ± 85.6 | <0.001*** |
| Pantothenic acid (mg) | 2.8 ± 1.4 | 5.6 ± 1.0 | <0.001*** |
| Biotin (μg) | 0.3 ± 1.0 | 18.3 ± 6.6 | <0.001*** |
| Calcium (mg) | 200.7 ± 118.7 | 816.5 ± 231.2 | <0.001*** |
| Iron (mg) | 8.0 ± 5.8 | 18.7 ± 4.8 | <0.001*** |
| Sodium (mg) | 1491.8 ± 813.6 | 1653.8 ± 682.1 | 0.030* |
| Zinc (mg) | 5.2 ± 2.3 | 18.3 ± 4.3 | <0.001*** |
| Cholesterol (mg) | 127.4 ± 148.6 | 152.6 ± 108.5 | 0.332 |
| Leucine (g) | 1.6 ± 0.9 | 5.8 ± 1.8 | <0.001*** |

Paired t-test: **P* < 0.05, ** *P* < 0.01, *** *P* < 0.001. Data are presented as the mean ± SD.

**Supplementary Table 3. Muscle function and biochemical parameters at baseline, and at Week 12 and 24**

| Parameters | Baseline | Week 12 | Baseline *vs*. week 12  *p* value | Week 24 | Week 12  *vs.* 24  *p* value | Baseline *vs*. Week 24  *p* value |
| --- | --- | --- | --- | --- | --- | --- |
| SMI (kg/m^2^) | 9.2 ± 2.5 | 9.3 ± 2.5 | 0.157 | 9.2 ± 2.3 | 0.430 | 0.851 |
| Fat mass (kg) | 20.3 ± 7.8 | 20.0 ± 8.1 | 0.529 | 19.2 ± 7.6 | 0.101 | 0.046* |
| BMI (kg/m^2^) | 23.9 ± 2.9 | 23.9 ± 3.1 | 0.283 | 23.7 ± 3.1 | 0.031* | 0.291 |
| HGS (kg) | 32.8 ± 9.9 | 32.8 ± 8.8 | 0.915 | 32.0 ± 9.4 | 0.060 | 0.197 |
| Gait speed (m/s) | 1.22 ± 0.29 | 1.35 ± 0.20 | 0.014* | 1.41 ± 0.21 | 0.043* | <0.001*** |
| 5TSTS (s) | 7.8 ± 2.9 | 6.5 ± 1.9 | 0.016* | 6.2 ± 1.8 | 0.262 | 0.003** |
| SPPB | 11.6 ± 0.7 | 12.0 ± 0.2 | 0.050* | 12.0 ± 0.2 | >0.999 | 0.031* |
| AST (U/L) | 14.1 ± 6.9 | 15.4 ± 6.7 | 0.190 | 12.9 ± 6.0 | 0.128 | 0.005** |
| ALT (U/L) | 13.3 ± 7.5 | 16.7 ± 7.6 | 0.006** | 13.1 ± 5.7 | 0.007** | 0.860 |
| ALP (U/L) | 79.1 ± 38.0 | 85.0 ± 33.6 | 0.314 | 74.7 ± 28.0 | 0.013* | 0.252 |
| TB (mg/dL) | 0.44 ± 0.21 | 0.41 ± 0.14 | 0.270 | 0.38 ± 0.13 | 0.223 | 0.020* |
| Total protein (g/dL) | 6.6 ± 0.4 | 6.7 ± 0.4 | 0.356 | 6.6 ± 0.4 | 0.297 | 0.972 |
| Albumin (g/dL) | 3.7 ± 0.3 | 3.8 ± 0.3 | 0.144 | 3.8 ± 0.3 | 0.638 | 0.380 |
| BUN (mg/dL) | 60.9 ± 16.2 | 73.9 ± 16.3 | 0.002** | 66.9 ± 14.0 | 0.100 | 0.177 |
| Cr (mg/dL) | 11.7 ± 4.2 | 11.6 ± 3.6 | 0.726 | 11.5 ± 4.3 | 0.952 | 0.872 |
| eGFR (ml/min/1.73m^2^) | 5.0 ± 4.2 | 4.7 ± 3.1 | 0.589 | 5.1 ± 4.1 | 0.682 | 0.947 |
| Calcium (mg/dL) | 8.6 ± 0.5 | 8.8 ± 0.8 | 0.279 | 8.8 ± 2.1 | 0.970 | 0.630 |
| Phosphorus (mg/dL) | 4.9 ± 1.0 | 5.0 ± 1.5 | 0.799 | 4.6 ± 1.6 | 0.192 | 0.242 |
| Uric acid (mg/dL) | 7.2 ± 1.4 | 7.5 ± 1.2 | 0.310 | 7.6 ± 1.4 | 0.979 | 0.464 |
| Glucose (mg/dL) | 131.1 ± 33.1 | 137.3 ± 32.9 | 0.337 | 138.6 ± 39.5 | 0.867 | 0.434 |
| Sodium (mEq/L) | 139.3 ± 2.70 | 138.4 ± 2.90 | 0.171 | 138.3 ± 2.80 | 0.868 | 0.017* |
| Potassium (mEq/L) | 4.8 ± 0.7 | 5.2 ± 0.6 | 0.041* | 4.9 ± 0.7 | 0.195 | 0.401 |
| Chloride (mEq/L) | 102.1 ± 3.50 | 101.1 ± 3.80 | 0.296 | 100.5 ± 3.90 | 0.438 | 0.020* |
| TC (mg/dL) | 147.0 ± 29.9 | 145.9 ± 27.3 | 0.734 | 145.3 ± 35.1 | 0.884 | 0.575 |
| TG (mg/dL) | 148.0 ± 70.5 | 167.9 ± 115.6 | 0.346 | 167.5 ± 95.2 | 0.984 | 0.249 |
| HDL (mg/dL) | 41.2 ± 12.3 | 44.6 ± 12.7 | 0.045* | 42.7 ± 11.8 | 0.085 | 0.333 |
| LDL (mg/dL) | 79.0 ± 25.9 | 72.7 ± 25.1 | 0.089 | 75.1 ± 31.2 | 0.532 | 0.226 |
| TCO_2_ (mmol/L) | 19.1 ± 3.6 | 20.2 ± 1.9 | 0.119 | 20.5 ± 1.9 | 0.423 | 0.054 |
| WBC (10^3^/μl) | 6.4 ± 1.6 | 6.4 ± 1.7 | 0.853 | 6.6 ± 1.2 | 0.494 | 0.678 |
| Hemoglobin (g/dL) | 10.3 ± 1.1 | 10.5 ± 1.0 | 0.624 | 10.2 ± 1.0 | 0.310 | 0.648 |
| Seg. neutrophil (%) | 66.6 ± 7.7 | 67.0 ± 8.7 | 0.845 | 66.7 ± 9.1 | 0.920 | 0.928 |
| Lymphocyte (%) | 21.9 ± 6.3 | 19.7 ± 6.2 | 0.104 | 20.7 ± 5.7 | 0.416 | 0.225 |
| Monocyte (%) | 7.4 ± 1.6 | 7.8 ± 2.1 | 0.339 | 6.8 ± 1.5 | 0.022* | 0.026* |
| MCV (fL) | 97.7 ± 4.3 | 97.1 ± 4.2 | 0.329 | 96.8 ± 4.2 | 0.622 | 0.192 |
| MCH (pg) | 32.4 ± 1.8 | 32.3 ± 1.3 | 0.435 | 32.6 ± 1.5 | 0.099 | 0.638 |
| MCHC (g/dL) | 33.2 ± 0.7 | 33.2 ± 0.7 | 0.859 | 33.6 ± 0.6 | 0.015* | 0.008** |
| MPV (fL) | 8.5 ± 0.9 | 8.3 ± 0.7 | 0.166 | 8.2 ± 0.7 | 0.487 | 0.037* |
| CRP (mg/dL) | 0.19 ± 0.36 | 0.12 ± 0.15 | 0.367 | 0.09 ± 0.10 | 0.135 | 0.124 |

Skeletal muscle index, SMI; Handgrip strength, HGS; Five times sit-to-stand test, 5TSTS; TB, Total bilirubin; TC, total cholesterol; TG, triglyceride; Seg. neutrophil, Segmented neutrophil. Repeated measures ANOVA with post-hoc Fisher’s LSD test: **P* < 0.05, ***P* < 0.01, ****P* < 0.001. Data are presented as the mean ± SD.

**Supplementary Table 4.** **Comparisons by sex in clinical factors including components of sarcopenia and serum biochemistry after 12 weeks of intervention.**

| Variables | Female (N=7) | Male (N=15) | *p* value |
| --- | --- | --- | --- |
| Age (years) | 57.7 ± 11.3 | 54.1 ± 12.2 | 0.520 |
| Height (cm) | 154.5 ± 4.1 | 171.9 ± 7.2 | <0.001*** |
| BSA (m^2^) | 1.53 ± 0.11 | 1.85 ± 0.13 | <0.001*** |
| Leucine intake (g/day) | 3.9 ± 2.0 | 4.3 ± 1.8 | 0.585 |
| Leucine intake per BSA (g/m^2^/day) | 2.5 ± 1.3 | 2.4 ± 1.0 | 0.779 |
| ΔBw (%) | 0.8 ± 1.2 | 0.1 ± 1.8 | 0.343 |
| HD duration (yrs) | 6.4 ± 4.2 | 3.9 ± 2.9 | 0.113 |
| ΔKt/V | 0.0 ± 0.2 | –0.1 ± 0.4 | 0.517 |
| Exercise compliance (%) | 81.6 ± 21.8 | 89.7 ± 11.1 | 0.379 |
| Leucine compliance (%) | 0.9 ± 0.2 | 0.7 ± 0.2 | 0.154 |
| Leucine side effects | 5 (71.4%) | 5 (33.3%) | 0.172 |
| Baseline HGS (kg) | 21.2 ± 2.9 | 38.2 ± 6.6 | <0.001*** |
| Grip responders | 5 (71.4%) | 2 (13.3%) | 0.014* |
| ΔHGS (%) | 7.6 ± 8.2 | –1.6 ± 7.2 | 0.015* |
| Baseline SMI (kg) | 17.5 ± 1.4 | 29 ± 4.9 | <0.001*** |
| SMI responders | 6 (85.7%) | 8 (53.3%) | 0.193 |
| ΔSMI (%) | 3.6 ± 3.7 | 0.6 ± 4.5 | 0.131 |
| Baseline gait speed (m/s) | 1.3 ± 0.2 | 1.2 ± 0.3 | 0.550 |
| Gait speed responders | 2 (28.6%) | 11 (73.3%) | 0.074 |
| ΔGait speed (%) | 7.3 ± 21.6 | 22.2 ± 35.8 | 0.324 |
| Baseline 5TSTS (s) | 7.6 ± 3.1 | 7.8 ± 2.9 | 0.881 |
| 5TSTS responder | 5 (71.4%) | 9 (60.0%) | 0.604 |
| Δ5TSTS (%) | –8.0 ± 24.3 | –11.8 ± 26.9 | 0.754 |
| ΔFat mass (%) | –3.4 ± 7.7 | –2.2 ± 12.6 | 0.826 |
| ΔTotal protein (g/dL) | 0.1 ± 0.4 | 0.1 ± 0.4 | 0.991 |
| ΔAlbumin (g/dL) | 0.1 ± 0.2 | 0.0 ± 0.2 | 0.422 |
| ΔBUN (mg/dL) | 16.4 ± 19.8 | 11.4 ± 16.4 | 0.541 |
| ΔCr (mg/dL) | 0.2 ± 1.2 | –0.2 ± 1.2 | 0.434 |
| ΔeGFR (ml/min/1.73m^2^) | 0.1 ± 0.9 | –0.5 ± 2.8 | 0.581 |
| ΔCa (mg/dL) | –0.3 ± 1.1 | 0.5 ± 0.7 | 0.070 |
| ΔP (mg/dL) | –0.6 ± 1.2 | 0.4 ± 0.9 | 0.058 |
| ΔUA (mg/dL) | 0.7 ± 1.1 | 0.2 ± 1.7 | 0.499 |
| ΔHemoglobin (g/dL) | 0.8 ± 1.1 | –0.1 ± 1.5 | 0.178 |

BSA, body surface area; Bw, body weight; HD, hemodialysis; HGS, hand-grip strength; SMI, skeletal muscle index; 5TSTS, five-times sit-to-stand test. Unpaired t-test and Chi-square test were used. Continuous variable data are shown in mean ± SD. Categorical variable data are shown in number and corresponding proportion. **P* < 0.05, ***P* < 0.01, ****P* < 0.001.

**Supplementary Table 5. Immunophenotypic characterization of PBMCs at baseline, and at Week 12 and 24**

| Parameters (%) | Baseline | Week 12 | Baseline *vs.* Week 12  p value | Week 24 | Week 12  *vs.* 24  p value | Baseline  *vs.* Week 24  p value |
| --- | --- | --- | --- | --- | --- | --- |
| CD4+ | 58.4 ± 14.1 | 59.6 ± 13.7 | 0.296 | 60.8 ± 15.1 | 0.569 | 0.317 |
| CD8+ | 32.6 ± 9.80 | 31.1 ± 8.90 | 0.182 | 30.0 ± 11.4 | 0.676 | 0.367 |
| CD4+CD57+ | 6.3 ± 5.7 | 7.7 ± 7.3 | 0.118 | 6.8 ± 6.8 | 0.532 | 0.735 |
| CD8+CD57+ | 34.9 ± 18.9 | 35.8 ± 18.1 | 0.450 | 35.4 ± 19.7 | 0.921 | 0.916 |
| CD14+CD16- | 69.0 ± 15.9 | 69.5 ± 15.8 | 0.779 | 65.7 ± 18.0 | 0.272 | 0.297 |
| CD14-CD16+ | 5.9 ± 3.7 | 5.6 ± 2.7 | 0.680 | 6.0 ± 4.1 | 0.667 | 0.908 |
| CD14+CD16+ | 6.8 ± 3.6 | 7.8 ± 6.6 | 0.234 | 8.3 ± 9.4 | 0.784 | 0.359 |
| CD3+CD56+ | 1.7 ± 2.3 | 1.4 ± 2.0 | 0.010** | 1.4 ± 1.8 | 0.907 | 0.216 |
| CD3+CD56- | 95.2 ± 5.30 | 95.5 ± 5.10 | 0.066 | 95.4 ± 5.0 | 0.852 | 0.611 |
| CD4+CD45RA+CD45RO+ | 0.8 ± 0.7 | 0.7 ± 0.8 | 0.448 | 0.7 ± 1.0 | 0.943 | 0.637 |
| CD4+CD45RA+CD45RO- | 43.0 ± 15.2 | 41.2 ± 15.2 | 0.078 | 40.4 ± 15.9 | 0.715 | 0.302 |
| CD4+CD45RA-CD45RO+ | 48.1 ± 14.9 | 47.8 ± 14.6 | 0.727 | 47.9 ± 14.8 | 0.975 | 0.922 |
| CD8+CD45RA+CD45RO+ | 0.2 ± 0.3 | 0.1 ± 0.1 | 0.225 | 0.1 ± 0.1 | 0.766 | 0.179 |
| CD8+CD45RA+CD45RO- | 45.5 ± 15.9 | 44.5 ± 17.0 | 0.247 | 45.1 ± 18.6 | 0.805 | 0.900 |
| CD8+CD45RA-CD45RO+ | 38.2 ± 14.1 | 36.3 ± 14.6 | 0.072 | 35.6 ± 15.7 | 0.793 | 0.383 |
| CD4+CD45RA+CD197+ | 0.8 ± 0.9 | 0.6 ± 0.7 | 0.121 | 0.6 ± 0.9 | 0.902 | 0.525 |
| CD4+CD45RA+CD279+ | 2.4 ± 2.6 | 2.4 ± 2.3 | 0.960 | 1.4 ± 1.9 | 0.073 | 0.126 |
| CD4+CD45RO+CD197+ | 1.4 ± 1.3 | 1.2 ± 1.2 | 0.160 | 1.2 ± 1.5 | 0.946 | 0.527 |
| CD4+CD45RO+CD279+ | 1.9 ± 3.4 | 1.6 ± 1.6 | 0.600 | 0.8 ± 1.2 | 0.050* | 0.051 |
| CD8+CD45RA+CD197+ | 8.3 ± 7.1 | 7.2 ± 6.7 | 0.140 | 7.1 ± 6.8 | 0.898 | 0.428 |
| CD8+CD45RA+CD279+ | 13.8 ± 9.10 | 14.0 ± 10.2 | 0.821 | 11.3 ± 8.00 | 0.072 | 0.110 |
| CD8+CD45RO+CD197+ | 2.0 ± 1.1 | 1.8 ± 1.4 | 0.461 | 1.3 ± 1.0 | 0.100 | 0.004** |
| CD8+CD45RO+CD279+ | 1.9 ± 1.6 | 2.1 ± 2.8 | 0.791 | 1.1 ± 1.2 | 0.122 | 0.032* |
| CD3+CD4+FOXP3+ | 4.7 ± 3.4 | 3.9 ± 3.2 | 0.286 | 3.7 ± 2.9 | 0.772 | 0.235 |
| CD4+CD197+TNFα+ | 2.0 ± 1.5 | 1.7 ± 1.6 | 0.291 | 1.2 ± 1.2 | 0.007** | 0.010* |
| CD4+CD45RO+TNFα+ | 4.3 ± 6.2 | 4.3 ± 6.0 | 0.943 | 4.2 ± 6.3 | 0.767 | 0.902 |
| CD4+CD45RO+IL17A+ | 1.0 ± 1.6 | 1.0 ± 1.1 | 0.987 | 0.7 ± 0.9 | 0.315 | 0.481 |
| CD4+CD197+IL17A+ | 0.7 ± 0.9 | 0.7 ± 1.1 | 0.782 | 0.2 ± 0.2 | 0.025* | 0.015* |
| γδTCR+TNFα+ | 10.3 ± 7.80 | 7.9 ± 5.2 | 0.052 | 7.5 ± 7.6 | 0.087 | 0.719 |
| γδTCR+IL17A+ | 2.6 ± 4.2 | 3.2 ± 4.6 | 0.237 | 2.0 ± 4.3 | 0.027* | 0.068 |
| CD8+CD45RO+TNFα+ | 2.3 ± 2.1 | 2.2 ± 2.3 | 0.839 | 2.0 ± 3.0 | 0.614 | 0.578 |
| CD8+CD197+TNFα+ | 2.1 ± 1.9 | 1.9 ± 1.8 | 0.615 | 1.4 ± 1.6 | 0.065 | 0.060 |
| CD8+CD57+TNFα | 1.1 ± 1.8 | 1.5 ± 2.6 | 0.509 | 0.7 ± 1.1 | 0.249 | 0.457 |
| CD8+CD57+INFγ+ | 3.0 ± 4.0 | 4.1 ± 4.2 | 0.035* | 1.6 ± 2.1 | 0.003** | 0.032* |

Repeated measure ANOVA with post-hoc Fisher’s LSD test: **P* < 0.05, ***P* < 0.01. Data are presented as the mean ± SD.

**Supplementary Table 6. Association between clinical variables and changes in muscle strength and physical performance parameters during the first 12 weeks**

| Parameters | Baseline parameter | Univariate regression | | Multivariate regression | |
| --- | --- | --- | --- | --- | --- |
|  |  | β ± SE | *p* value | β ± SE | *p* value |
| ΔHandgrip  Strength | Sex (ref. male) | -9.165 ± 3.451 | 0.015 |  |  |
|  | Height | -0.503 ± 0.146 | 0.003 |  |  |
|  | Body weight | -0.335 ± 0.149 | 0.037 |  |  |
|  | ASM | -0.748 ± 0.223 | 0.003 |  |  |
|  | Baseline handgrip strength | -0.534 ± 0.153 | 0.002 | -0.493 ± 0.101 | 0.000 |
|  | Exercise compliance | 10.042 ± 3.009 | 0.003 | 6.752 ± 2.034 | 0.004 |
|  | CD8+ | 0.401 ± 0.174 | 0.032 |  |  |
|  | CD4+CD45RA+CD45RO+ | -5.610 ± 2.426 | 0.032 |  |  |
|  | CD8+CD45RA+CD45RO+ | -16.651 ± 5.942 | 0.011 |  |  |
|  | CD8+CD45RO+CD279+ | 2.864 ± 1.143 | 0.023 |  |  |
|  | CD8+CD57+TNFα+ | -2.270 ± 0.952 | 0.027 | -1.805 ± 0.578 | 0.006 |
| ΔGait speed | Baseline Gait speed | -12.584 ± 2.277 | <0.001 | -10.517 ± 2.305 | 0.000 |
|  | Triglyceride | -0.130 ± 0.058 | 0.036 |  |  |
|  | Neutrophil | 1.196 ± 0.522 | 0.033 |  |  |
|  | Monocyte | -5.593 ± 2.546 | 0.040 |  |  |
|  | MCHC | -16.216 ± 5.130 | 0.005 | -8.607 ± 3.999 | 0.044 |
| ΔFive times sit-to-stand test | Baseline 5TSTS | -5.453 ± 1.570 | 0.002 | -5.276 ± 1.373 | 0.001 |
|  | BMI | -4.116 ± 1.732 | 0.028 |  |  |
|  | Monocyte | -6.817 ± 3.224 | 0.047 |  |  |
|  | CD14+CD16- | -0.643 ± 0.331 | 0.066 |  |  |
|  | CD4+CD45RO+CD279+ | 3.819 ± 1.458 | 0.016 |  |  |
|  | CD8+CD45RO+CD279+ | 10.908 ± 4.132 | 0.018 | 6.679 ± 2.488 | 0.015 |

HGS, hand-grip strength; ASM, appendicular skeletal muscle mass; MCHC, mean corpuscular hemoglobin concentration; 5TSTS, five time sit-to-stand; BMI, body mass index.

**Supplementary Table 7. Markers of T cell senescence in ASM non-responders and responders**

|  | ASM | Baseline | Week 12 | Baseline *vs*. Week 12  p value | Week 24 | Week 12  *vs.* 24  p value | Baseline *vs*. Week 24  p value |
| --- | --- | --- | --- | --- | --- | --- | --- |
| CD4/CD8 | NR | 2.1 ± 0.9 | 2.4 ± 1.2 | 0.129 | 2.4 ± 0.9 | 0.989 | 0.242 |
|  | RES | 2.0 ± 0.9 | 2.0 ± 0.8 | 0.877 | 2.2 ± 1.0 | 0.236 | 0.148 |
| CD14+CD16+ | NR | 7.1 ± 4.2 | 7.9 ± 6.0 | 0.867 | 10.6 ± 11.9 | 0.397 | 0.325 |
|  | RES | 6.6 ± 3.4 | 7.8 ± 7.2 | 0.215 | 6.9 ± 7.8 | 0.325 | 0.896 |
| CD4+CD57+ | NR | 7.3 ± 6.1 | 8.4 ± 7.3 | 0.575 | 5.6 ± 5.6 | 0.156 | 0.452 |
|  | RES | 5.8 ± 5.6 | 7.3 ± 7.5 | 0.093 | 7.5 ± 7.5 | 0.905 | 0.366 |
| CD8+CD57+ | NR | 36.3 ± 22.4 | 37.5 ± 20.0 | 0.454 | 34.3 ± 19.7 | 0.693 | 0.815 |
|  | RES | 34.1 ± 17.4 | 34.9 ± 17.6 | 0.665 | 36.0 ± 20.4 | 0.850 | 0.724 |
| CD8+CD57+TNFα | NR | 0.3 ± 0.6 | 1.7 ± 2.8 | 0.195 | 0.8 ± 1.7 | 0.476 | 0.468 |
|  | RES | 1.5 ± 2.1 | 1.3 ± 2.5 | 0.857 | 0.6 ± 0.7 | 0.393 | 0.188 |
| CD8+CD57+INFγ | NR | 2.8 ± 2.8 | 4.8 ± 4.0 | 0.130 | 1.3 ± 1.2 | 0.028^#^ | 0.150 |
|  | RES | 3.2 ± 4.6 | 3.7 ± 4.4 | 0.120 | 1.8 ± 2.6 | 0.053 | 0.135 |
| CD4+CD45RA+CD45RO- | NR | 53.2 ± 7.5* | 53.4 ± 7.8** | 0.897 | 48.2 ± 15.2 | 0.149 | 0.234 |
|  | RES | 37.1 ± 15.5 | 34.2 ± 14.1 | 0.034^#^ | 36.0 ± 15.0 | 0.481 | 0.719 |
| CD4+CD45RA-CD45RO+ | NR | 38.2 ± 7.5* | 37.6 ± 8.5** | 0.629 | 42.7 ± 18.2 | 0.212 | 0.353 |
|  | RES | 53.8 ± 15.2 | 53.6 ± 14.3 | 0.912 | 50.9 ± 12.3 | 0.264 | 0.352 |
| CD4+CD45RA+CD197+ | NR | 0.7 ± 0.8 | 0.6 ± 0.4 | 0.226 | 0.9 ± 1.2 | 0.510 | 0.804 |
|  | RES | 0.8 ± 1.0 | 0.6 ± 0.8 | 0.274 | 0.5 ± 0.6 | 0.552 | 0.251 |
| CD4+(CD45RA+/CD45RO+) | NR | 1.5 ± 0.5* | 1.5 ± 0.5** | 0.697 | 1.5 ± 1.0* | 0.759 | 0.972 |
|  | RES | 0.8 ± 0.6 | 0.7 ± 0.5 | 0.101 | 0.8 ± 0.4 | 0.576 | 0.765 |
| CD8+CD45RA+CD45RO- | NR | 52.4 ± 16.7 | 50.0 ± 16.6 | 0.076 | 48.6 ± 18.0 | 0.722 | 0.342 |
|  | RES | 41.6 ± 14.5 | 41.4 ± 17.1 | 0.878 | 43.2 ± 19.3 | 0.620 | 0.687 |
| CD8+CD45RA-CD45RO+ | NR | 30.4 ± 11.9* | 30.6 ± 12.3 | 0.886 | 31.6 ± 16.2 | 0.750 | 0.756 |
|  | RES | 42.7 ± 13.6 | 39.6 ± 15.2 | 0.019^#^ | 37.9 ± 15.6 | 0.263 | 0.684 |
| CD8+CD45RA+CD197+ | NR | 10.1 ± 8.00 | 9.1 ± 7.9 | 0.347 | 8.3 ± 6.5 | 0.732 | 0.532 |
|  | RES | 7.2 ± 6.6 | 6.2 ± 5.9 | 0.271 | 6.4 ± 7.1 | 0.900 | 0.645 |
| CD8+(CD45RA+/CD45RO+) | NR | 2.3 ± 1.9 | 2.0 ± 1.3 | 0.480 | 2.3 ± 1.9 | 0.397 | 0.927 |
|  | RES | 1.2 ± 0.8 | 1.4 ± 1.1 | 0.067 | 1.5 ± 1.2 | 0.508 | 0.134 |

ASM, appendicular skeletal muscle mass; NR, non-responder; RES, responder. Repeated measure ANOVA with post-hoc Fisher’s LSD test: *Comparison between NR and RES; ^#^Comparison between different time points. **P* < 0.05, ***P* < 0.01, ^#^*P* < 0.001. Data are presented as the mean ± SD.
