# Supplementary material for: Leucine-enriched amino acid supplementation and exercise to prevent sarcopenia in patients on hemodialysis: a single-arm pilot study
Source: Front Nutr. 2023 Apr 28;10:1069651. doi: 10.3389/fnut.2023.1069651 (PMC10176607; doi:10.3389/fnut.2023.1069651)
Supplement: Supplementary file 2 [file Presentation_1.PPTX]

## Slide 1
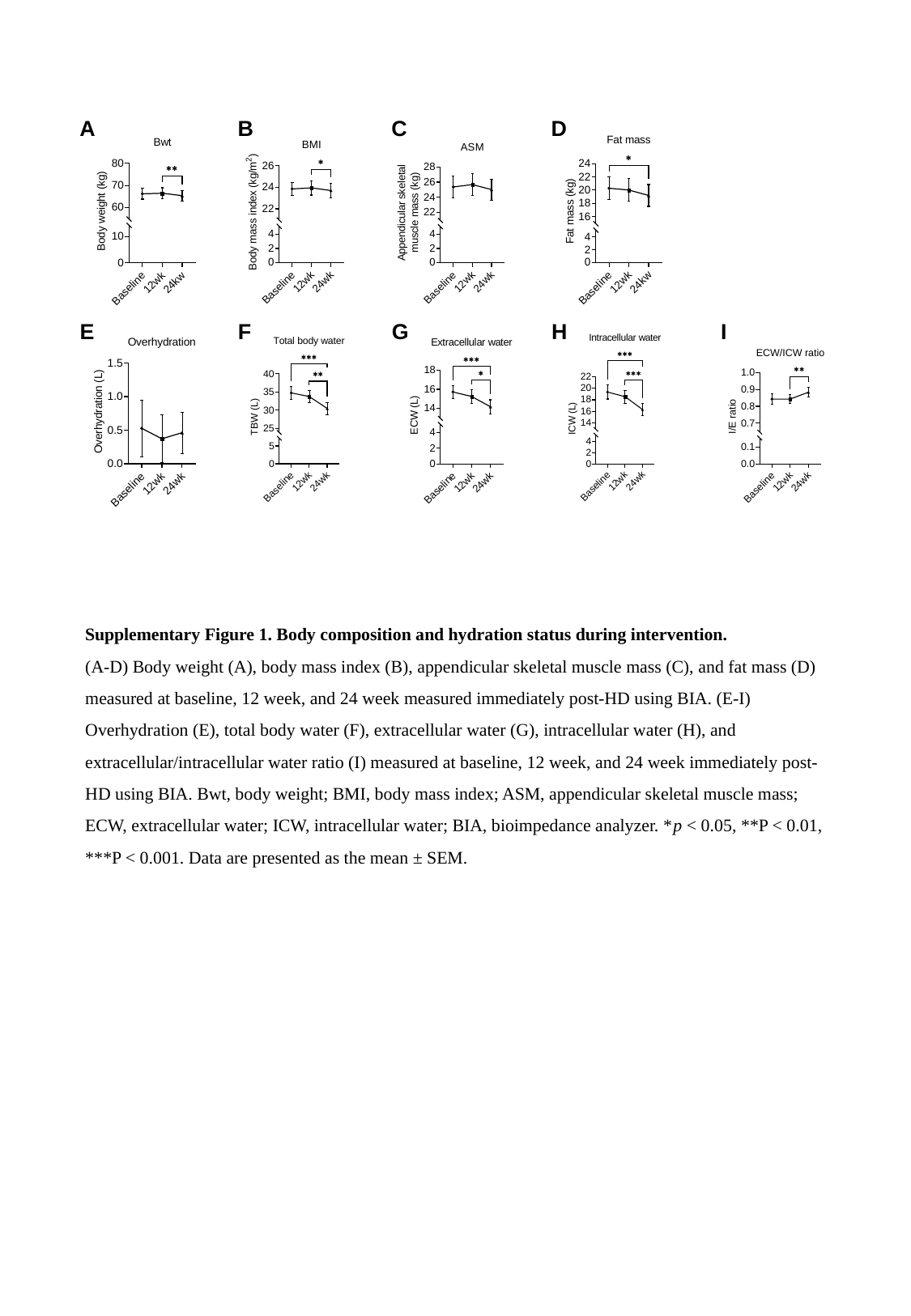

Supplementary Figure 1. Body composition and hydration status during intervention.
(A-D) Body weight (A), body mass index (B), appendicular skeletal muscle mass (C), and fat mass (D) measured at baseline, 12 week, and 24 week measured immediately post-HD using BIA. (E-I) Overhydration (E), total body water (F), extracellular water (G), intracellular water (H), and extracellular/intracellular water ratio (I) measured at baseline, 12 week, and 24 week immediately post-HD using BIA. Bwt, body weight; BMI, body mass index; ASM, appendicular skeletal muscle mass; ECW, extracellular water; ICW, intracellular water; BIA, bioimpedance analyzer. *p < 0.05, **P < 0.01, ***P < 0.001. Data are presented as the mean ± SEM.

## Slide 2
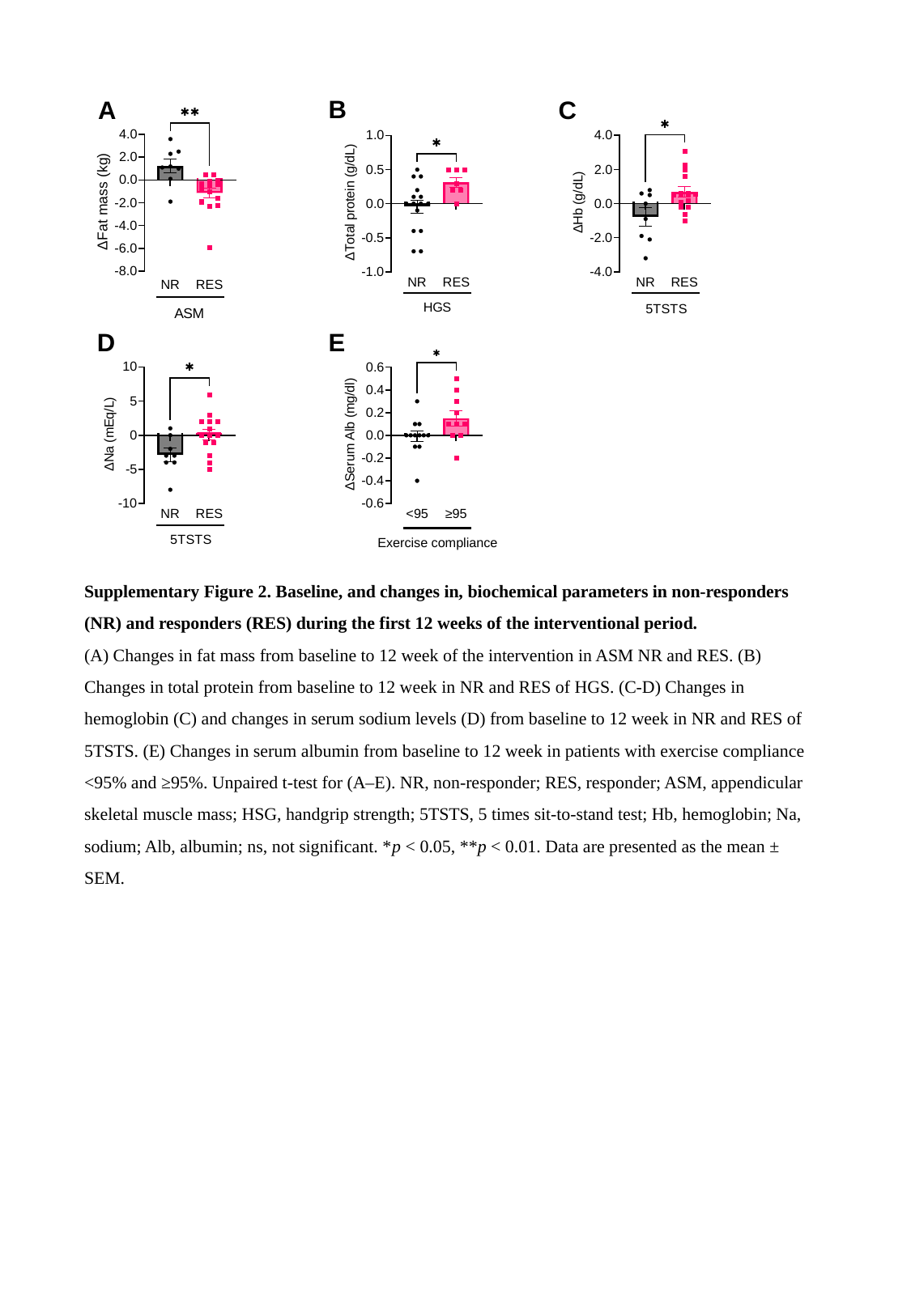

Supplementary Figure 2. Baseline, and changes in, biochemical parameters in non-responders (NR) and responders (RES) during the first 12 weeks of the interventional period.
(A) Changes in fat mass from baseline to 12 week of the intervention in ASM NR and RES. (B) Changes in total protein from baseline to 12 week in NR and RES of HGS. (C-D) Changes in hemoglobin (C) and changes in serum sodium levels (D) from baseline to 12 week in NR and RES of 5TSTS. (E) Changes in serum albumin from baseline to 12 week in patients with exercise compliance <95% and ≥95%. Unpaired t-test for (A–E). NR, non-responder; RES, responder; ASM, appendicular skeletal muscle mass; HSG, handgrip strength; 5TSTS, 5 times sit-to-stand test; Hb, hemoglobin; Na, sodium; Alb, albumin; ns, not significant. *p < 0.05, **p < 0.01. Data are presented as the mean ± SEM.

## Slide 3
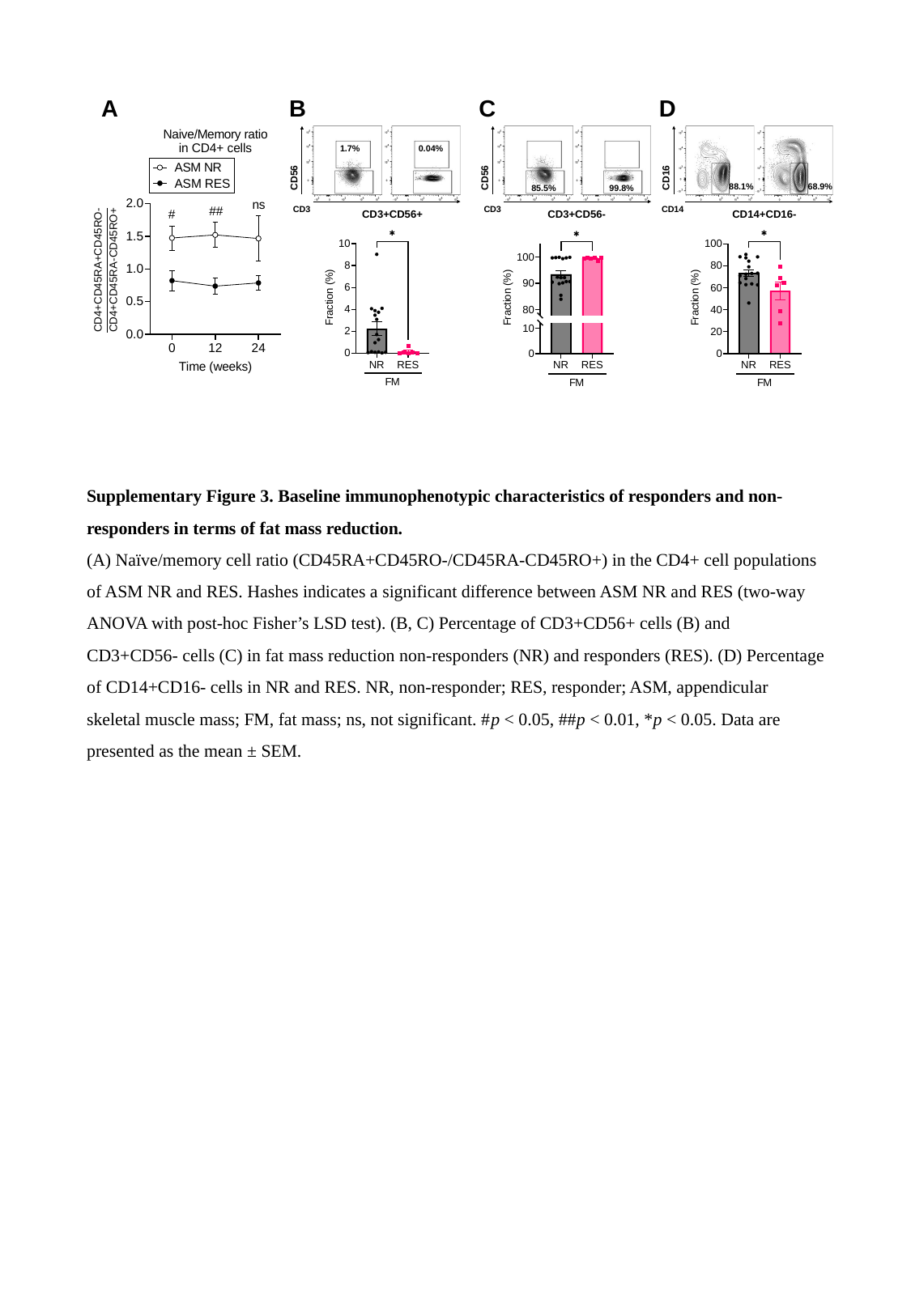

Supplementary Figure 3. Baseline immunophenotypic characteristics of responders and non-responders in terms of fat mass reduction.
(A) Naïve/memory cell ratio (CD45RA+CD45RO-/CD45RA-CD45RO+) in the CD4+ cell populations of ASM NR and RES. Hashes indicates a significant difference between ASM NR and RES (two-way ANOVA with post-hoc Fisher’s LSD test). (B, C) Percentage of CD3+CD56+ cells (B) and CD3+CD56- cells (C) in fat mass reduction non-responders (NR) and responders (RES). (D) Percentage of CD14+CD16- cells in NR and RES. NR, non-responder; RES, responder; ASM, appendicular skeletal muscle mass; FM, fat mass; ns, not significant. #p < 0.05, ##p < 0.01, *p < 0.05. Data are presented as the mean ± SEM.

## Slide 4
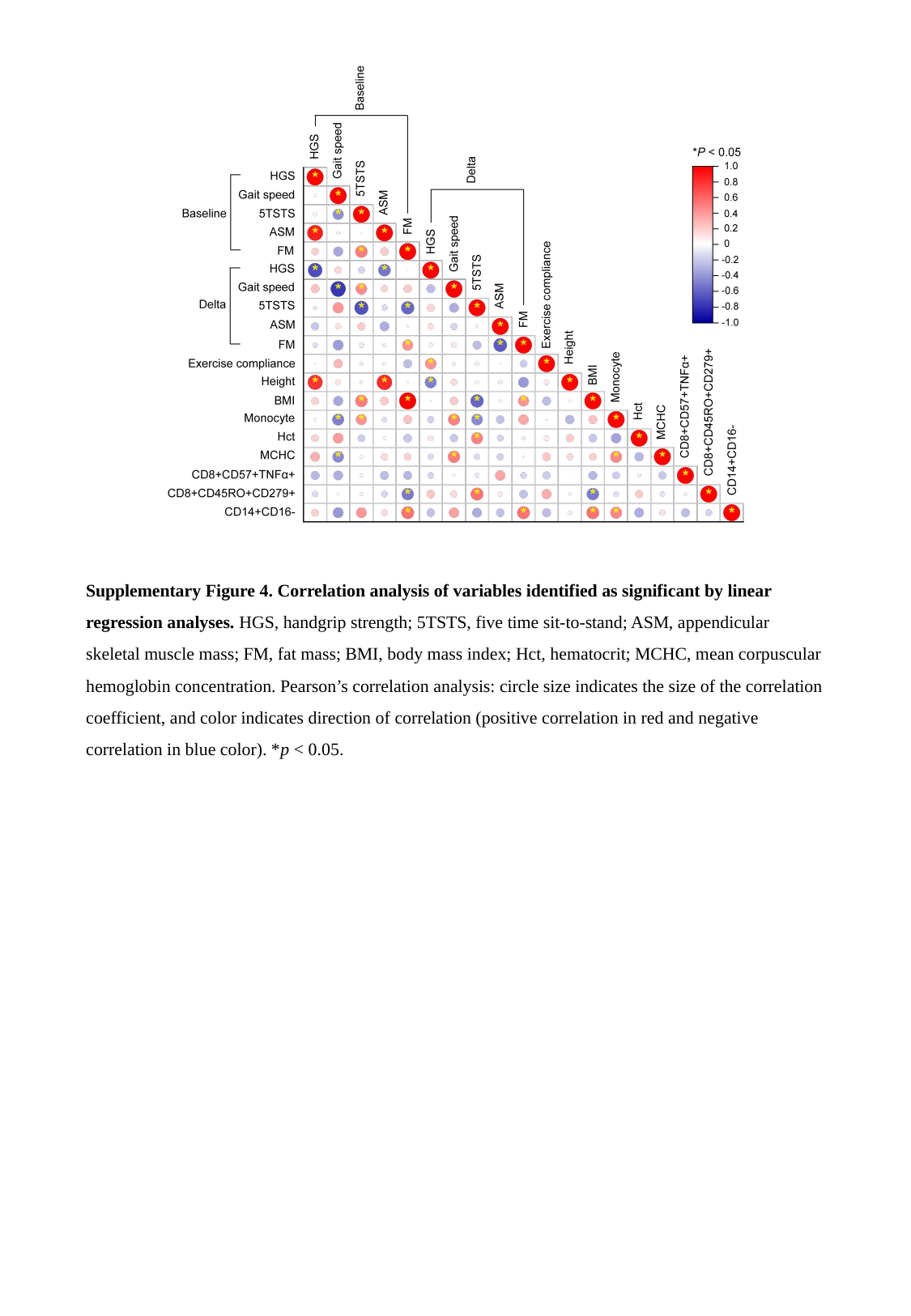

Supplementary Figure 4. Correlation analysis of variables identified as significant by linear regression analyses. HGS, handgrip strength; 5TSTS, five time sit-to-stand; ASM, appendicular skeletal muscle mass; FM, fat mass; BMI, body mass index; Hct, hematocrit; MCHC, mean corpuscular hemoglobin concentration. Pearson’s correlation analysis: circle size indicates the size of the correlation coefficient, and color indicates direction of correlation (positive correlation in red and negative correlation in blue color). *p < 0.05.

## Slide 5
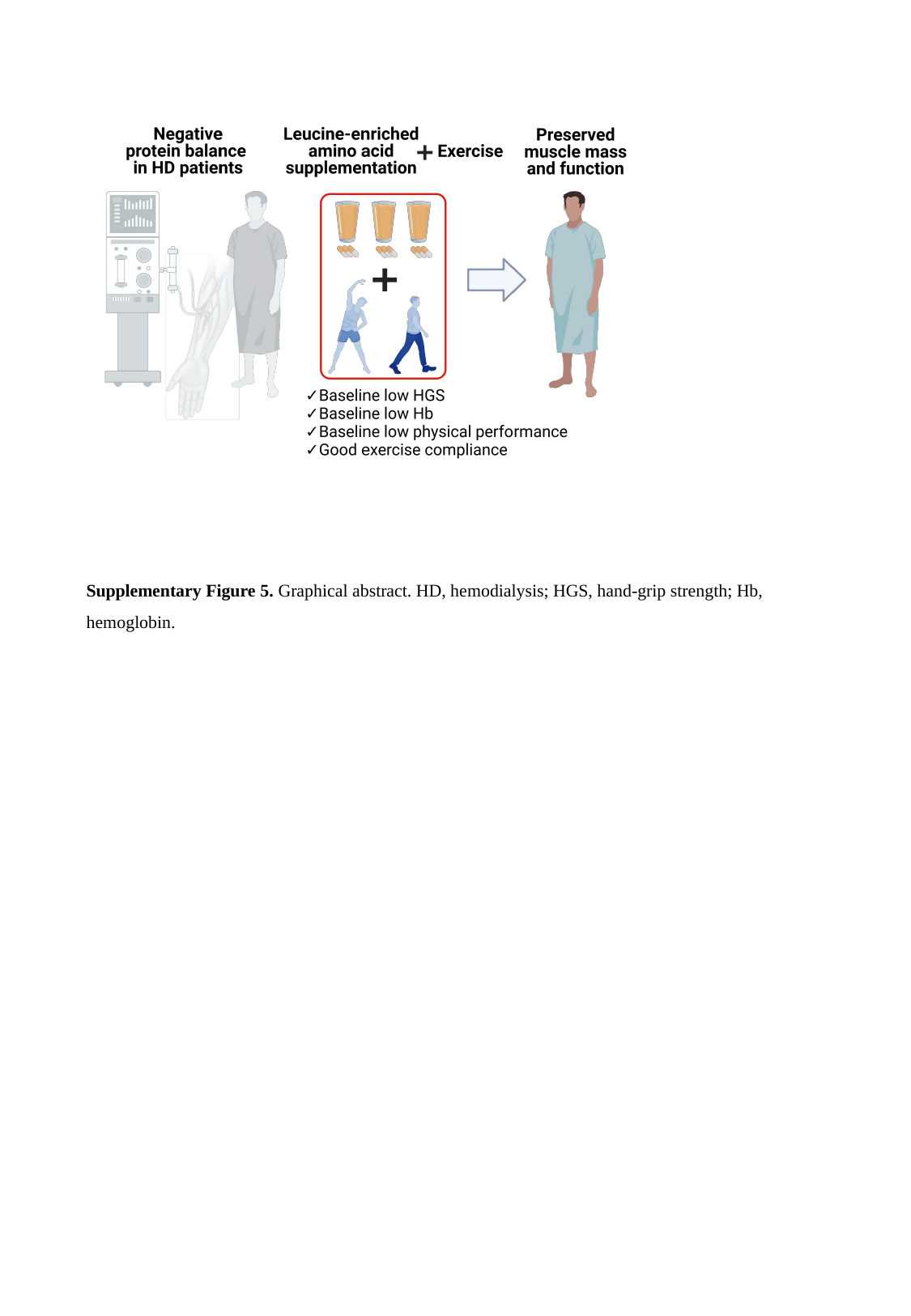

Supplementary Figure 5. Graphical abstract. HD, hemodialysis; HGS, hand-grip strength; Hb, hemoglobin.
