# Supplementary material for: Leucine-enriched amino acid supplementation and exercise to prevent sarcopenia in patients on hemodialysis: a single-arm pilot study
Source: Front Nutr. 2023 Apr 28;10:1069651. doi: 10.3389/fnut.2023.1069651 (PMC10176607; doi:10.3389/fnut.2023.1069651)
Supplement: Supplementary file 3 [file Data_Sheet_1.PDF]

Stretching

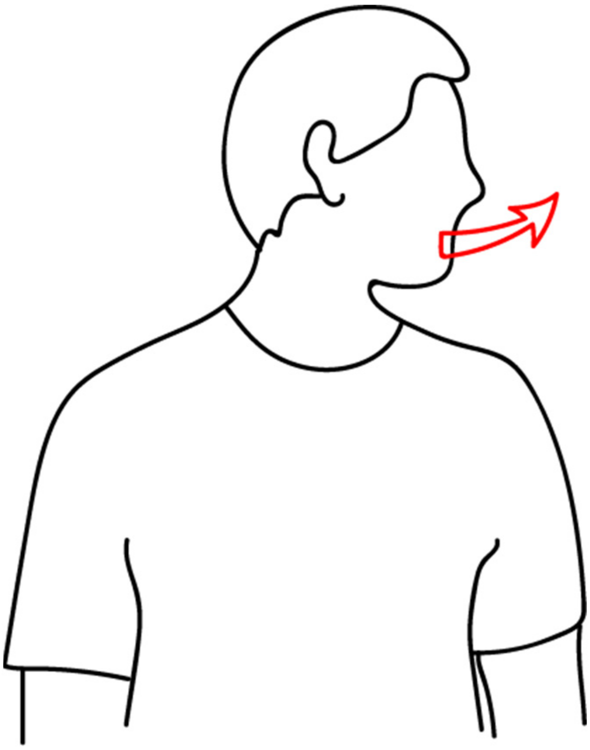

1 Neck rotation

Turn your head to look over your right shoulder. Hold for 10 seconds.
Repeat with other side.

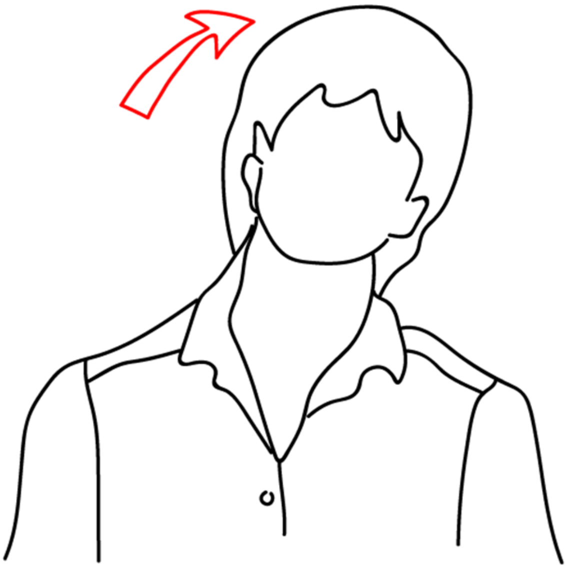

2 Tilt to the side of the neck

Put your hand over your head and slowly bring it down to your shoulder. Hold for 10 seconds.
Repeat with the other side.

Stretching

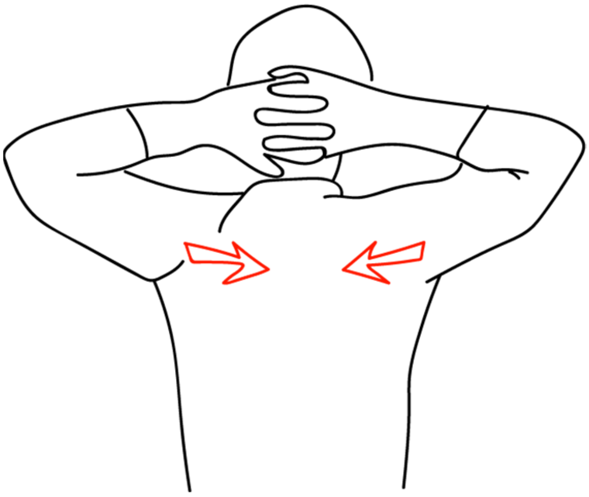

3 Stretch your chest

Put your fingers behind your neck and open your chest with your elbows behind you. Stop for 10 seconds.

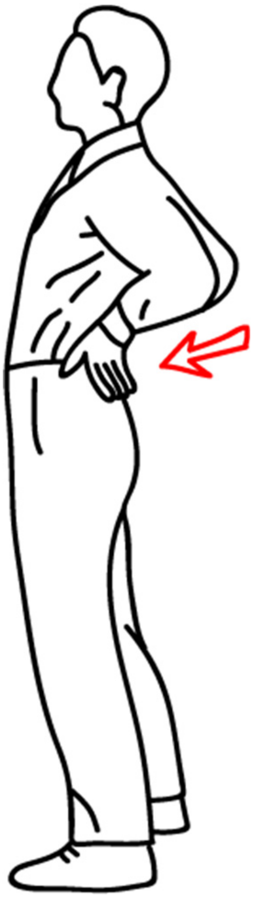

4 Anterior Chest Stretch

Place your palms on your lower back just above your hip. Finger pointing downward. Gently push your palms forward to create an extension in the lower back. Hold for 10 seconds.

Stretching

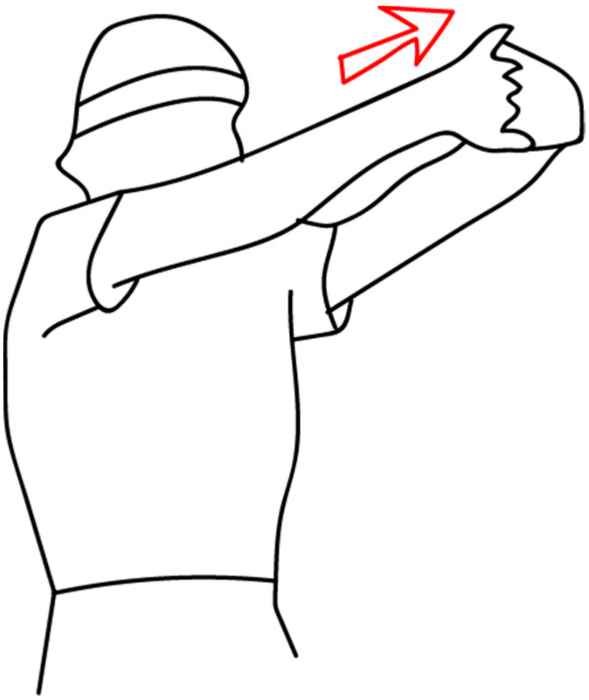

5 Stretch your arms forward

Interlock fingers of both hands at shoulder height with your palms facing away from your body. Keep elbows straight. Hold for 10 seconds

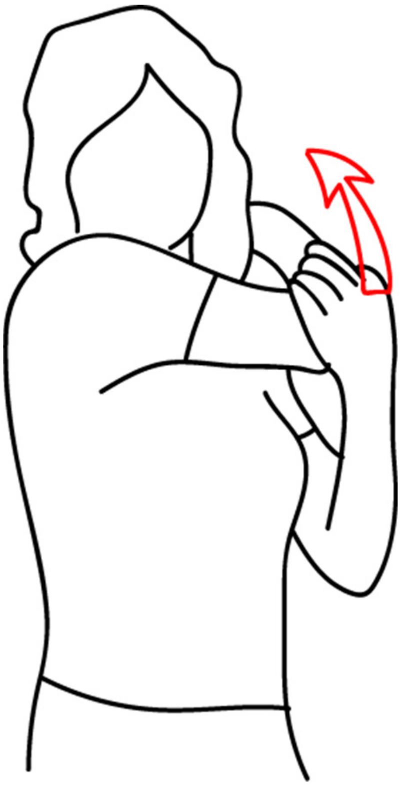

6 Horizontal Shoulder Stretch

Raise your left arm horizontally and hold your elbow with your right hand. Pull back for 10 seconds. Repeat with left hand on right shoulder. Pull back for 10 seconds.

Stretching

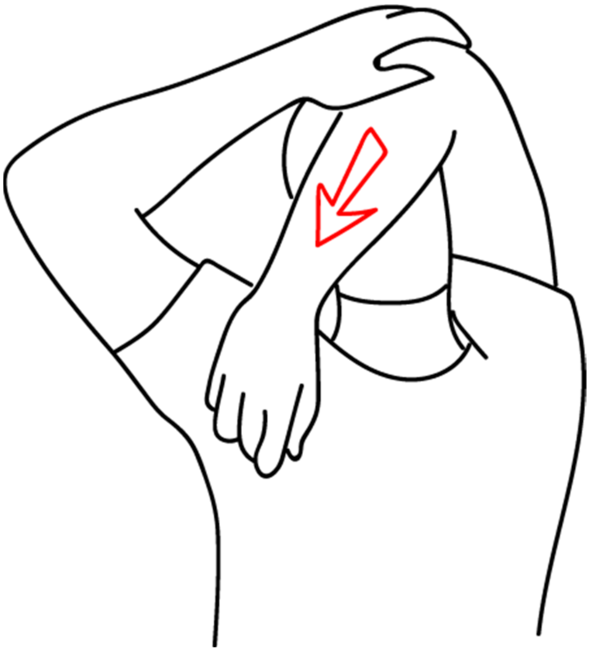

7 Overhead tricep stretch

Bring right arm across your body and over your left shoulder, holding your elbow with your left hand. Press for 10 seconds. Then repeat for your other arm

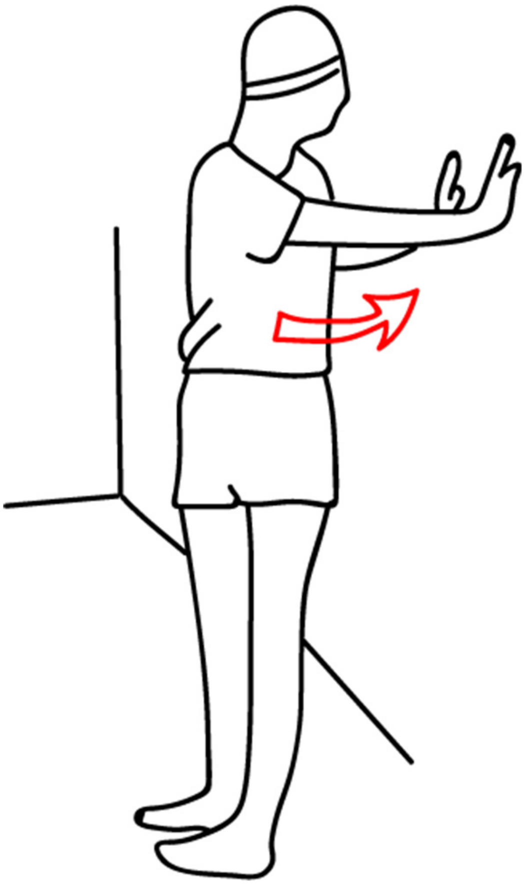

8 Turn left and right the body

Stand near the wall, turn your body to the left, and attach your palm to the wall. Stop for 10 seconds. The opposite direction is the same.

Arm exercise

2 bottles of 500 ml water/ 1 set 10 times, 3 sets in total

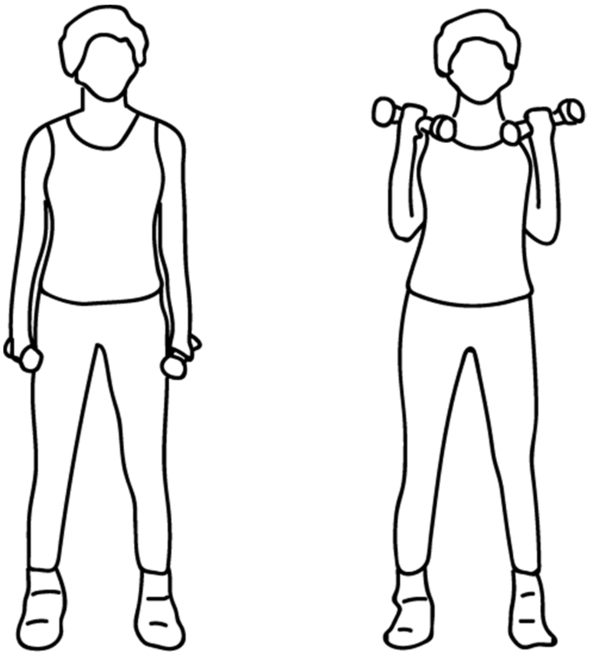

1 Pulling your arms towards your body

Lift a dumbbell(Water bottle) and pull your arms toward your body.
Perform 3 sets of 10 times per set.

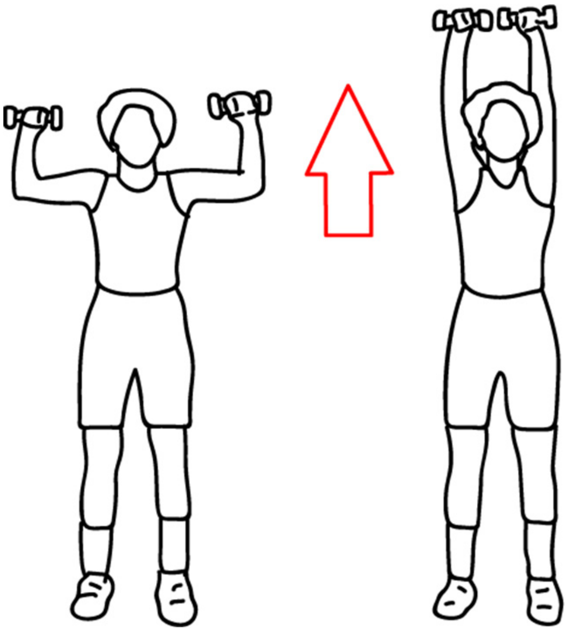

2 Stretch out your arms

Hold a dumbbell (water bottle) and straighten your elbows at right angles.
Perform 3 sets of 10 times per set.

Arm exercise

2 bottles of 500 ml water/ 1 set 10 times, 3 sets in total

※ Caution: Exercise safely in a chair with a backrest and without wheels.

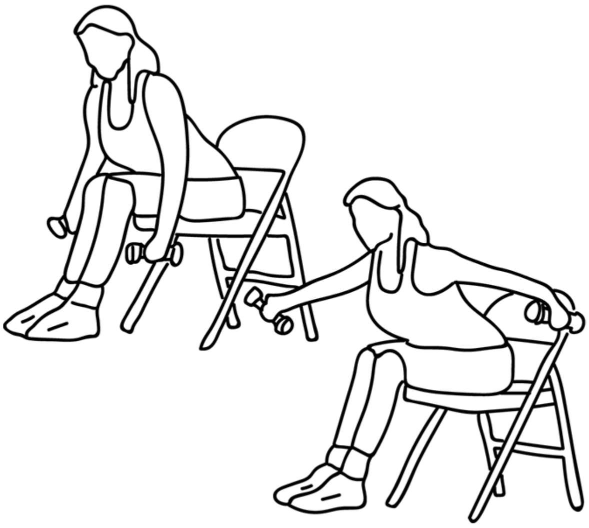

3 Raising one's arms horizontally

Sit on a chair with a dumbbell (water bottle) and spread your arms horizontally.
Perform 3 sets of 10 times per set.

Leg exercise

Chair / 1 set 10 times, 3 sets in total

※ Caution: Exercise safely in a chair with a backrest and without wheels.

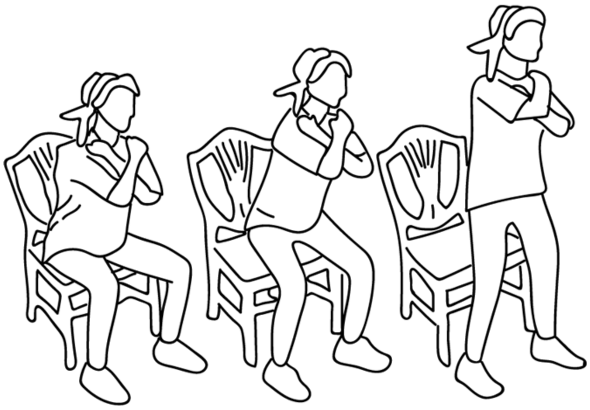

1 Sit down in a chair Stand up

Put both arms together on your chest, sit on a chair, and stand up.
Perform 3 sets of 10 times per set.

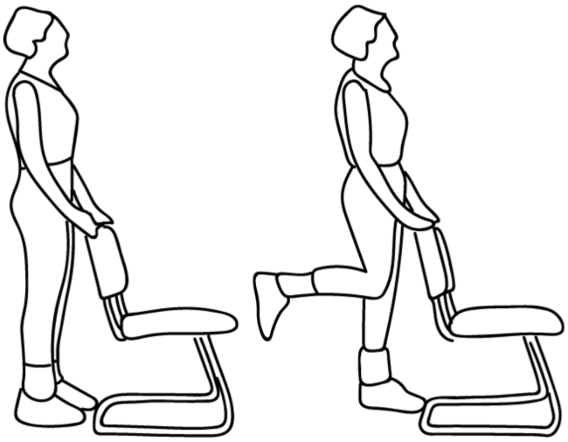

2 The back of one's foot

Stand behind the chair and grab the chair. Hold your left foot back for 5 seconds. Repeat 3 times. The other leg is also implemented.

Leg exercise

Chair / 1 set 10 times, 3 sets in total

※ Caution: Exercise safely in a chair with a backrest and without wheels.

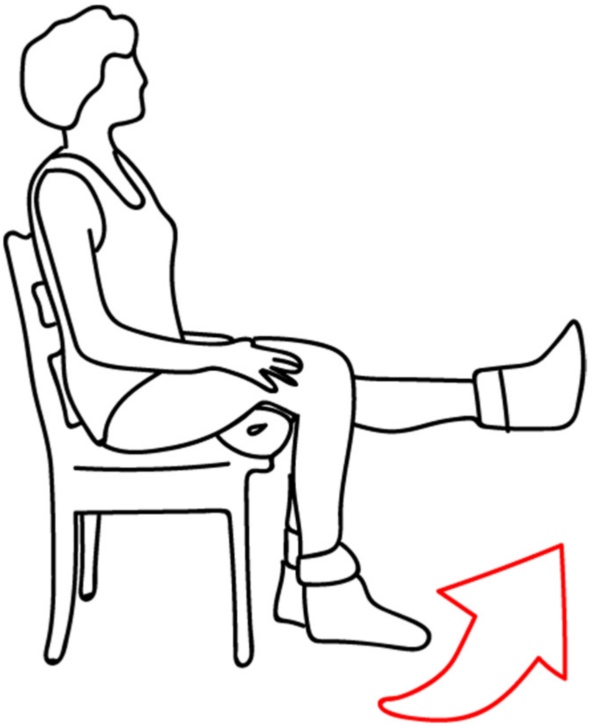

3 The front of one's feet

Hold your left foot forward for 5 seconds while sitting on a chair. Repeat three times. After that, the opposite foot does the same.

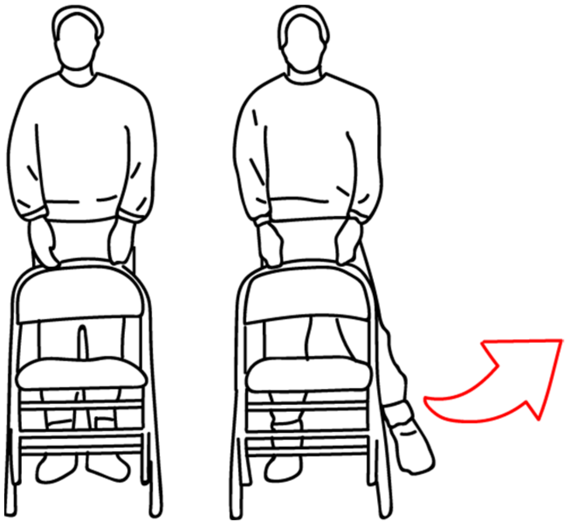

4 Lift your feet to the side

Stand behind the chair and grab the chair. Hold your left foot sideways for 5 seconds. Repeat 3 times. The other foot is carried out in the same way.

Leg exercise and walking

※ Caution: Exercise safely in a chair with a backrest and without wheels.

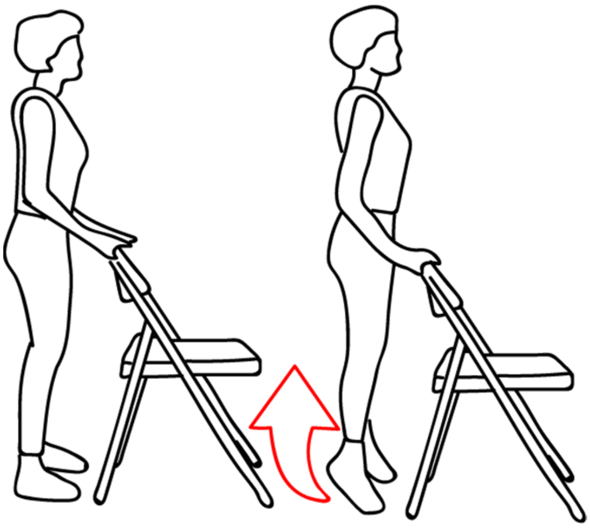

5 A lift in the heel

Stand behind the chair and grab the chair. Hold your heels for 5 seconds. Repeat three times.

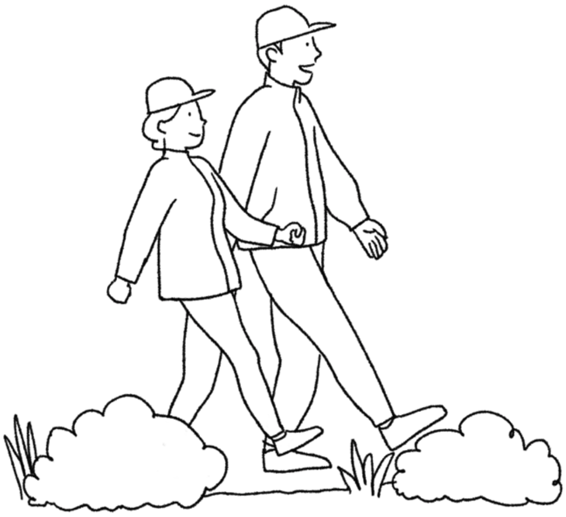

1 Walking

Walk for 20 minutes every day with a little fast pace.
